# Conformational Changes in Talin on Binding to Anionic Phospholipid Membranes Facilitate Signaling by Integrin Transmembrane Helices

**DOI:** 10.1371/journal.pcbi.1003316

**Published:** 2013-10-31

**Authors:** Antreas C. Kalli, Iain D. Campbell, Mark S. P. Sansom

**Affiliations:** Department of Biochemistry, University of Oxford, Oxford, United Kingdom; Tel Aviv University, Israel

## Abstract

Integrins are heterodimeric (αβ) cell surface receptors that are activated to a high affinity state by the formation of a complex involving the α/β integrin transmembrane helix dimer, the head domain of talin (a cytoplasmic protein that links integrins to actin), and the membrane. The talin head domain contains four sub-domains (F0, F1, F2 and F3) with a long cationic loop inserted in the F1 domain. Here, we model the binding and interactions of the complete talin head domain with a phospholipid bilayer, using multiscale molecular dynamics simulations. The role of the inserted F1 loop, which is missing from the crystal structure of the talin head, PDB:3IVF, is explored. The results show that the talin head domain binds to the membrane predominantly via cationic regions on the F2 and F3 subdomains and the F1 loop. Upon binding, the intact talin head adopts a novel V-shaped conformation which optimizes its interactions with the membrane. Simulations of the complex of talin with the integrin α/β TM helix dimer in a membrane, show how this complex promotes a rearrangement, and eventual dissociation of, the integrin α and β transmembrane helices. A model for the talin-mediated integrin activation is proposed which describes how the mutual interplay of interactions between transmembrane helices, the cytoplasmic talin protein, and the lipid bilayer promotes integrin inside-out activation.

## Author Summary

Transmission of signals across the cell membrane is an essential process for all living organisms. Integrins are one example of cell surface receptors (αβ) which, uniquely, form a bidirectional signalling pathway across the membrane. Integrins are crucial for many cellular processes and play key roles in pathological defects such as cardiovascular diseases and cancer. They are activated to a high affinity state by the intracellular protein talin in a process known as ‘inside-out activation’. Despite their importance and the existence of functional and structural data, the mechanism by which talin activates integrin remains elusive. In this study we use a multi-scale computational approach, which combines coarse-grained and atomistic molecular dynamics simulations, to suggest how the formation of the complex between the talin head domain, the cell membrane and the integrin moves the integrin equilibrium towards an active state. Our results show that conformational changes within the talin head domains optimize its interactions with the cell membrane. Upon binding to the integrin, talin facilitates rearrangement of the integrin TM region thus promoting integrin activation. This study also provides a demonstration of the strengths of a computational multi-scale approach in studies of membrane interactions and receptor conformational changes and associated proteins that enable transmembrane signaling.

## Introduction

Integrins are cell surface receptors involved in many essential cellular processes, such as cell migration, and in pathological defects, such as thrombosis and cancer [1]. Integrins are αβ heterodimers. Each subunit has a large ectodomain, a single transmembrane (TM) helix and a short flexible cytoplasmic tail [2]. Integrins are crucial for many signal transduction events [3]–[6]. Unusually, they can transmit signals in both directions across the cell membrane [2], [7]. In the inside-out activation pathway, formation of a complex between talin, the integrin β cytoplasmic tail, and the membrane is thought to shift the integrin conformational equilibrium towards an active state [8]–[15]. Activation is believed to proceed via changes in TM helix packing [2], [3] coupled to substantive conformational changes in the integrin ectodomain.

**Figure 1:**
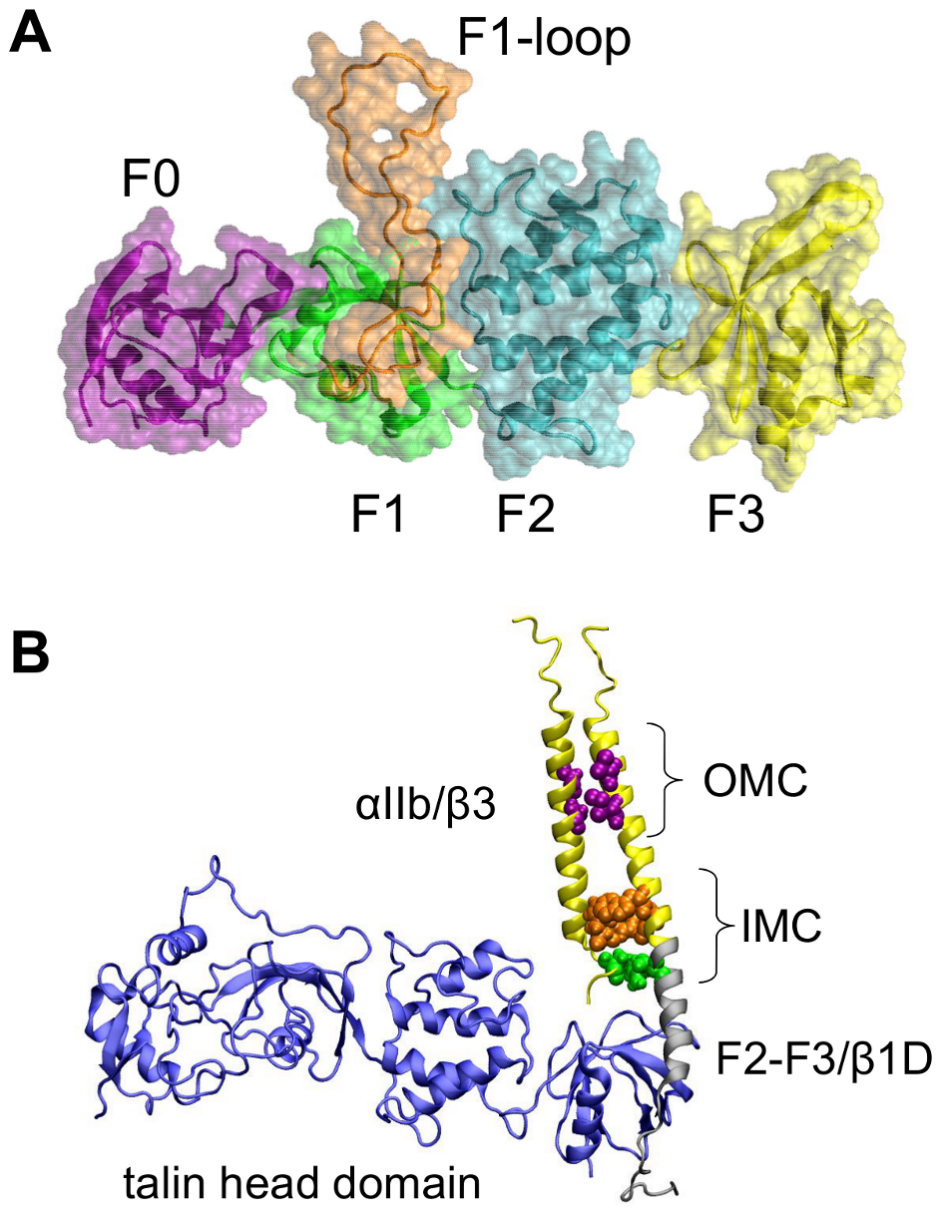
Talin and the talin/integrin TM complex. A. Structure of talin (F0–F3; PDB:3IVF), showing the F0 domain in purple, the F1 domain in green, the F1 insertion in orange, the F2 domain in cyan, and the F3 domain in yellow. The loop inserted in the F1 domain was generated using Modeller. B. Model of the talin/αβ complex. The β chimeric peptide was comprised of β3 residues 688–719/β1D residues 753–787. The part of the structure corresponding to the αIIb/β3 structure (PBD:2K9J) is shown in yellow, the F2–F3/β1D (PDB:3G9W) is in grey, and the talin head domain (PDB:3IVF) is in blue.

Talin consists of a head domain (∼50 kDa) and a large rod domain (∼220 kDa) [16], [17]. A crystal structure of the talin head domain revealed a novel linear arrangement of four subdomains, F0, F1, F2 and F3 (Fig. 1A). Although the head domain has sequence homology with other FERM (band four-point-one, ezrin, radixin, moesin) domains [18], the linear arrangement of the subdomains, an inserted loop in the F1 domain, and the extra F0 domain confer on talin significantly different features from canonical FERM domains [19]. Experimental studies of the talin head have yielded valuable insights into the role of the different subdomains [8], [12], [17], [20], [21]. A key step in integrin activation is binding of the F3 subdomain to the integrin β tail [9], [10], [12]–[14]. The other subdomains have also been shown to contribute to integrin activation, although they do not bind directly to the integrin β subunit [8]. The F2 subdomain has a positively charged patch which has been shown to enhance activation by interacting with negatively charged lipid headgroups in the membrane [11], [21]. The F0 and F1 subdomains have an ubiquitin-like fold [17], [20] along with a flexible, positively charged loop of ∼40 residues inserted in the F1 domain. This loop (which was removed to facilitate structure determination of the head domain [17]) has been proposed to form a transient helix stabilized by interactions with acidic phospholipids [20]. Structural studies of the talin head domain and fragments have also suggested that the F2–F3 and the F0–F1 domain pairs are relatively rigid but the pairs are connected by a flexible linker [17].

The inactive state of integrins is in part maintained by interactions between the α and β TM helices. Important interactions are defined by regions known as the outer (OMC) and inner (IMC) membrane clasps (Fig. 1B). In the OMC, a GxxxG motif in the α integrin TM region allows close packing with the β integrin helix whereas in the IMC there are hydrophobic interactions involving a cluster of phenylalanines and a salt bridge between the α and β chains [22]. Various models for TM helix rearrangements, including ‘scissor’ or ‘piston’ movements and increased helix separation have been proposed to explain activation and trans-membrane signaling [10], [23]–[29] but there remains a need to distinguish and refine these models.

Molecular dynamics (MD) simulations allow us to explore the conformational dynamics and lipid interactions of membrane proteins [30]. Multiscale approaches combine coarse-grained molecular dynamics (CG-MD) simulations [31], [32], which extend the time scales that can be studied, with subsequent all-atom simulations, which allow refinement of the system [33]. We previously used this approach to develop a model that explained how the F2–F3 fragment of the talin head domain associates with the plasma membrane in a way that led to a scissoring movement of the two integrin TM helices [34]. In the current study, we build upon these studies to elucidate the interactions between the *complete* talin head domain (i.e. domains F0–F3), a lipid bilayer, and the α/β integrin TM regions. Our results provide novel information about the orientation of the intact talin head in complex with the lipid bilayer, and about changes induced in the TM helical regions. Overall, the results reveal how binding of talin to the membrane and to integrins tails leads to integrin activation.

## Results/Discussion

### Overview of the Simulations

We have used a serial multiscale MD simulation [35] approach to explore the dynamics of the talin head (F0–F3) domain, its interaction with lipid bilayers, and the resultant conformational changes of a talin/integrin TM complex embedded in a phospholipid bilayer. This approach has previously been used to explore the interactions of a number of peripheral proteins with membrane surfaces and lipids [11], [36]–[39], of TM helices within a lipid bilayer [40], [41], and of more complex signaling and related assemblies within membranes [34]. It enables one to combine coarse-grained (CG) simulations of membrane association and related events, with more detailed atomistic simulations to refine the resultant models. It thus provides a complementary approach to extended atomistic simulations [42].

**Figure 2:**
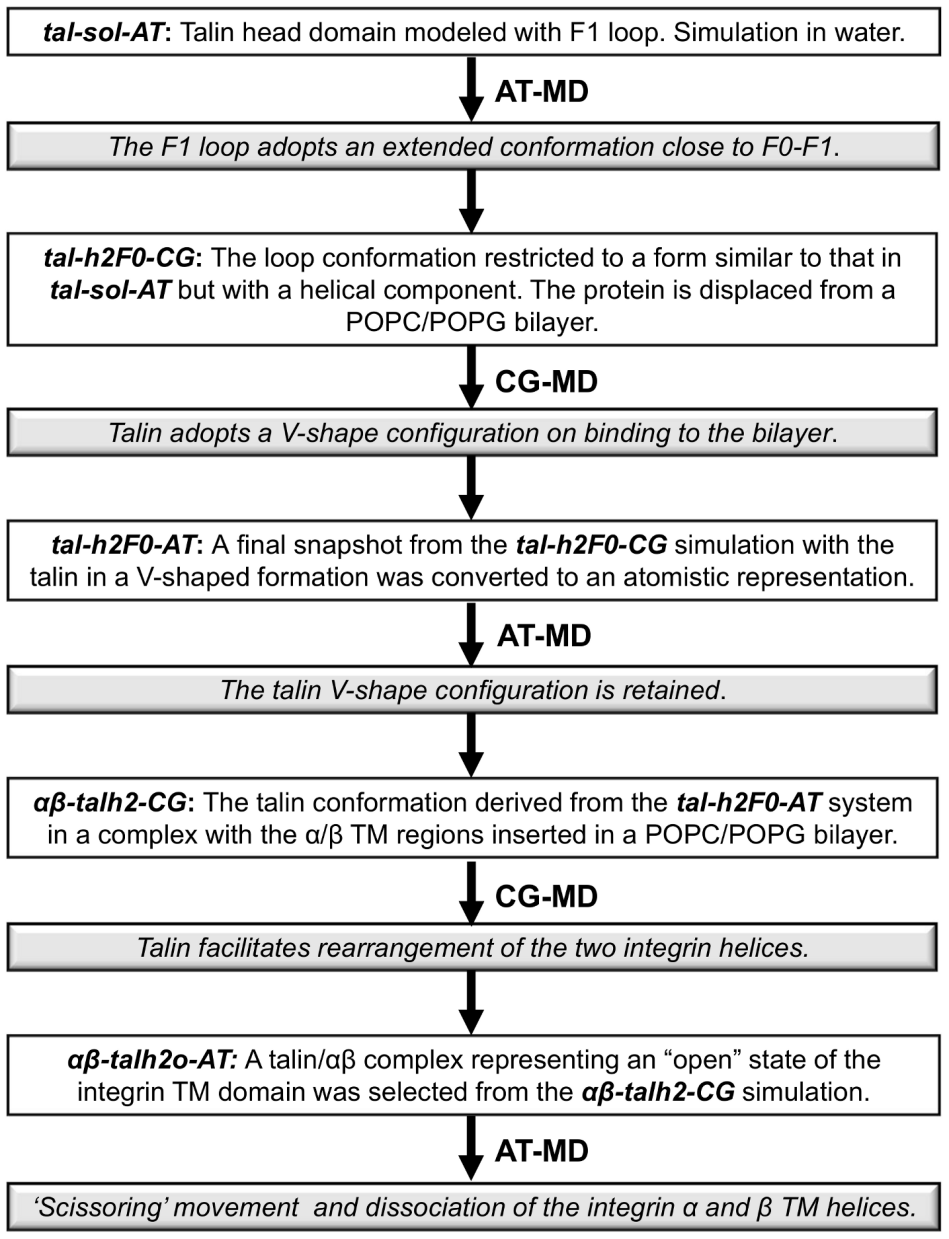
Flowchart of the principal simulations. Schematic representation of the inputs (white background boxes) and outputs (grey background boxes) of the simulations performed. See Table 1 for further information.

**Figure 3:**
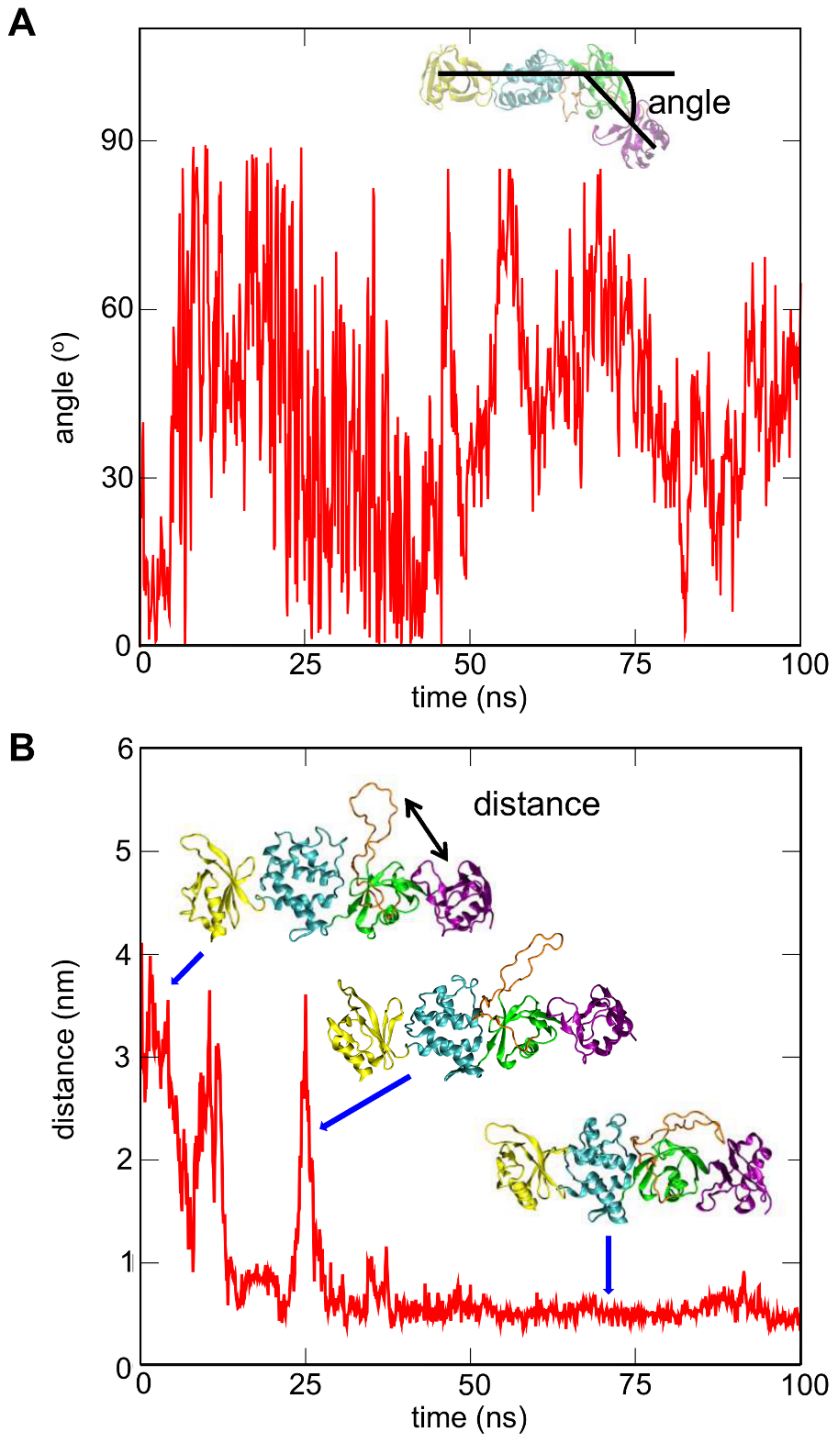
Simulation of talin in water (*tal-sol-AT*; see **Table 1**). A. Change in the angle of the F0–F1 pair relative to the F2–F3 pair from its initial position (i.e. the crystal structure) during a simulation in aqueous solution. The definition of the angle is shown in the inset. For this calculation two vectors were used: vector one was defined from the center of mass of the backbone particles of the F3 subdomain to the center of mass of the backbone particles of the F2 242–248/283–295 residue region. Similarly, vector two was defined from the center of mass of the backbone particles of the F0 subdomain to the center of mass of the backbone particles of F2 (residues 242–248/283–295). The angle displayed is the difference between the angle formed by these two vectors in the crystal structure and the angle formed by the two vectors in each snapshot of the simulation. B. Distance between the F1 loop (residue: L145) and the talin F0 domain (residue: G11) during the same simulation. The position of the loop is shown in the inset pictures at different time points.

The principal simulations underlying the current study are summarized in Table 1 and in Fig. 2. The talin head domain crystal structure (PDB:3IVF [17]) lacks a long loop region in the F1 domain and therefore atomistic simulations of the talin head domain (*tal-sol-AT*) in solution were first used to explore potential internal flexibility between the four component sub-domains (F0–F3) along with possible conformations of the F1 domain insertion. For these simulations the insertion in the F1 domain (res: 134–172) was modeled in a random coil conformation, and was located away from the F0–F1 pair (see Fig. 3B and Fig. S1A) using Modeller 9v8 (http://salilab.org/modeller/) [43], [44]. This configuration of the loop allows exploration of all possible conformations/orientations and selection of a preferred conformation/orientation relative to the talin head domain.

**Table 1:** Summary of the principal simulations.

Simulation	Description	Duration
tal-sol-AT	talin in water; AT-MD	2×0.1 µs
tal-h2F0-CG	talin association with an anionic bilayer; CG-MD	5×1.5 µs
tal-h2F0-AT	talin association with an anionic bilayer; AT-MD	3×45
αβ-talh2-CG	αβ TM + talin in a bilayer; CG-MD	5×4 µs
αβ-talh2o-AT	αβ TM + talin in a bilayer; AT-MD	1.0 µs

This table provides of the principal simulations; further details of these and of all other simulations are provides in Supporting Information Tables S1 and S2 and in Fig. 2.

The conformation of the talin head domain suggested by the above simulations was used to model the association of the talin head domain with a phospholipid bilayer. Since NMR studies suggested a helical propensity for the region involving residues 154–167 [20] this region was modeled as an α-helix (*tal-h2F0-CG* and Fig. S1). Subsequently, the same multiscale simulation approach was used to explore the dynamic behavior of a talin/TM integrin complex in a bilayer (*αβ-talh2-CG*) on a multi-microsecond timescale. The talin head domain/αβ complex was constructed as described in Kalli *et al.* [34] using the αΙΙbβ3 TM region NMR structure [22], the F2–F3/β1D complex crystal structure [12] and the talin head domain configuration obtained from the talin head domain simulations described in this study. An ‘open’ model generated by these CG simulations was subsequently explored via a microsecond duration atomistic simulation (*αβ-talh2o-AT*).

A number of control simulations were also performed to evaluate the robustness/sensitivity of the results and to explore the contributions of different regions and interactions (e.g. flexibility within the domain, electrostatic interactions and other helical conformations in the F1 loop) to the binding of the talin head to anionic lipid bilayers. Detailed descriptions of these simulations are provided in the Supporting Information (Tables S1 and S2). In total our study amounts to ca. 60 µs of CG-MD and ca. 2 µs of atomistic molecular dynamics simulations (AT-MD) simulation time.

### Talin Head Domain Dynamics in Solution

To study the conformational dynamics of the talin head domain prior to the association with the bilayer atomistic (AT-MD) simulations of the talin head domain (i.e. subdomains F0 to F3) in aqueous solution in the absence of a bilayer were performed (*tal-sol-AT* in Table 1). During these simulations the flexible linker between the F2–F3 and F0–F1 pairs allowed transient displacement of the F0–F1 subdomain relative to the F2–F3 subdomain with the angle defined in Fig. 3A. This angle, equal to 0° for a linear arrangement of F0-F1-F2-F3 as seen in the crystal structure, ranged from 0° to 90° in the simulations. During these simulations the long loop in the F1 domain moved closer to the F0–F1 pair, and adopted an extended conformation on the same side of the protein as the positively charged patch on F2 (Fig. 3B). Calculation of the electrostatic field around the talin head conformation observed at the end of these simulations suggests that localization of this loop close to the F0–F1 pair creates an extensive positively charged surface on one side of protein; this could facilitate strong talin/bilayer interactions (Fig. S2). Despite experimental evidence for an α-helical propensity in the F1 loop [17], no helix formation was detected (this might be due to insufficient simulation time for a coil-to-helix conformational transition to occur). Although there was a relatively large change in the angle *between* the F0–F1 and F2–F3 domain pairs, no significant angle change was observed *within* either the F0–F1 or the F2–F3 domain pair, suggesting that each pair behaves approximately as a rigid body.

### Interactions of the Talin Head Domain (F0–F3) with a Lipid Bilayer

**Figure 4:**
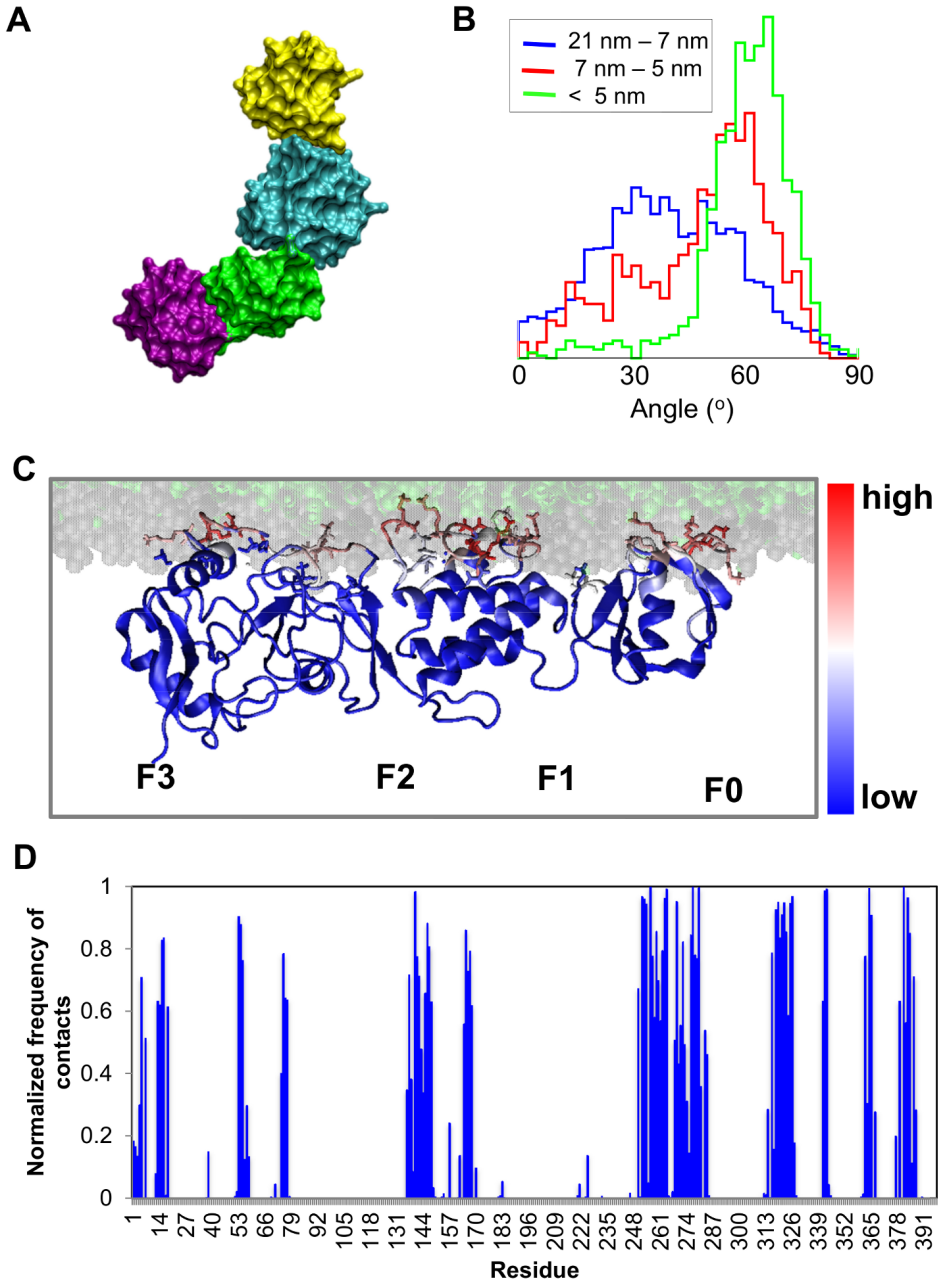
Simulation of the association of talin with a lipid bilayer (*tal-h2F0-CG*; seeTable 1). A. Final snapshot from the *tal-h2F0-CG* simulation, illustrating how the F0–F1 pair (purple-green) has been displaced relative to the F2–F3 pair (cyan-yellow) resulting in a V-shaped conformation. B. Change in the angle between the F0–F1 and the F2–F3 domain pairs as a function of distance from the bilayer during the *tal-h2F0-CG* simulation. The diagram shows the probability of finding an angle between the F0–F1 and the F2–F3 pairs at three different regions away from the bilayer phosphate atoms. C, D. Normalized number of contacts between the talin and lipids mapped onto the final snapshot (C) of the *tal-h2F0-CG* simulations. Contacts are defined by using a distance cut-off of 7 Å between the protein residues and the lipids. Blue indicates a low number (i.e zero contacts) white indicates a medium number (i.e. 7500 contacts) and red a large number of contacts (i.e. 15000 contacts). The bilayer headgroups are shown as grey spheres and the lipid tails as green spheres. The sidechains of the key basic residues (i.e. ARG and LYS), which are in contact with the lipids, are also shown. The residues that made more than 90% of the contacts during the tal-h2F0-AT simulations are shown in Table S3.

**Figure 5:**
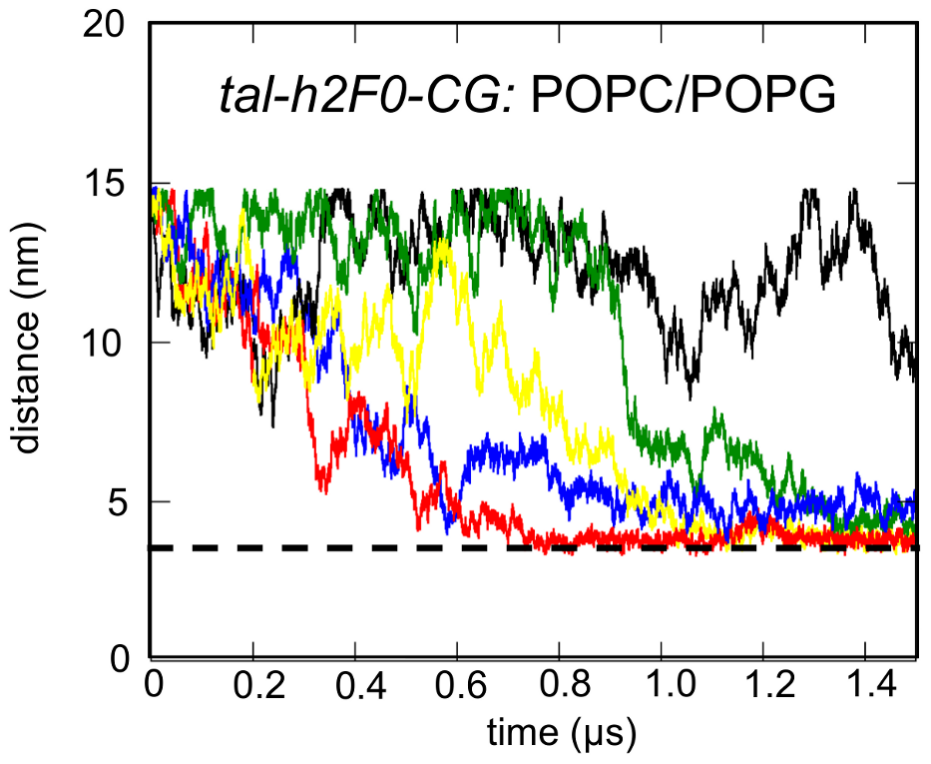
Simulation of the association of talin with a lipid bilayer (*tal-h2F0-CG*; see Table 1). Distance between the centers of mass of talin and a lipid bilayer as a function of time for the simulation *tal-h2F0-CG* with an anionic (POPC/POPG) lipid bilayer. The different colored lines correspond to the five repeat simulations. The horizontal broken line indicates the distance observed when talin is associated with the bilayer surface.

Having established in the *tal-sol-AT* simulations (see above) that the F1 loop interacts with F0–F1 to form a positively charged surface that extends the positive patch on F2–F3, CG simulations with the loop in this location were performed to explore the nature of the interactions of the complete talin head domain with an anionic lipid bilayer. Note that in this simulation system a small helical region (h2 helix; see Fig. S1) was included within the F1 loop as indicated by NMR data [20]. During this modeling of the loop the remainder of the structure, with the exception of the region modeled as helical (res: 154–167), was restrained to maintain the talin conformation derived from the above simulations. These restraints were removed during the simulations. In the *tal-h2F0-CG* simulation (Table 1; Fig. 4 and Fig. 5), talin was observed to associate with the bilayer in four out of five simulation and in all four of these simulations talin bound to the bilayer initially via a basic loop (res: 318–330) in the F3 domain, and subsequently via the positively charged patch in the F2 domain (res: 255–285) (Fig. S3A). Contacts were defined by using a distance cut-off of 7 Å between the protein residues and the lipids. These two regions have been identified previously [11] to promote productive binding of the isolated F2–F3 fragment to an anionic lipid bilayer. The additional surface created by the F1 loop also interacted with the bilayer (Fig. 4C). Interestingly, in all simulations which resulted in a talin/bilayer complex, the talin head domain with the F1 loop adopted a V-shaped conformation due to rotation of the F0–F1 pair relative to the F2–F3 pair in the bilayer plane (Fig. 4A). The reorientation of the F2–F3 and F0–F1 domain pairs prior to the binding to the bilayer was also observed in other simulations in which a different starting conformation of talin was used (e.g. the talin crystal structure; data not shown). In contrast to the more dynamic variations in the angle between the F0–F1 and F2–F3 observed when talin was in solution (see above), the V-shaped conformer was stabilized by association with the bilayer (Fig. 4B). This conformation optimizes talin/lipid interactions and induces a more compact arrangement of domains, although this new arrangement is still different from the linear arrangement in the X-ray structure [17] and the canonical FERM domain packing of F0 to F3 [18]. During the AT-MD simulations (*tal-h2F0-AT*; see Table 1) that started from the final snapshot of the *tal-h2F0-CG* simulation, the V-shaped conformation of talin was retained, with talin interacting preferentially with the headgroups of the anionic POPG lipids (Fig. S6B). No restrictions in the position/flexibility of the loop or the domains were imposed in the AT-MD simulations. Simulations of the talin head domain with a neutral bilayer (containing 1-palmitoyl-2-oleoyl-sn-glycero-3-phosphatidyl-choline (POPC) lipids) resulted in no association of talin with the bilayer (Fig. S3B and Text S1). These results are in good agreement with the available experimental data [11], [12], [17] and augment our previous observation that electrostatic interactions are important in regulating the formation of a talin/membrane complex.

Control CG simulations starting with the talin head domain crystal structure (i.e. without the F1 loop) with the same POPC/POPG (1-palmitoyl-2-oleoyl-sn-glycero-3-phosphatidyl-choline/1-palmitoyl-2-oleoyl-sn-glycero-3-phosphatidyl-glycerol) bilayer showed that the positively charged region of F0–F1 augmented by the F1 loop promotes direct F0–F1/bilayer interactions (data not shown). CG models of talin with restricted flexibility between the F0–F1 and F2–F3 domain pairs did not bind to the bilayer in a way that would facilitate binding of F2–F3 to the β-tail in any of these simulations (see Text S1). Disruption of the electrostatic interactions between talin and the membrane (simulating with experimentally tested mutations [10], [12]) also resulted in the ‘non-productive’ orientation of the talin head domain relative to the membrane (see Methods for description of mutations). An orientation is judged here to be ‘non-productive’ when the talin/bilayer complex formed is incompatible with binding to the β integrin cytoplasmic region in a manner similar to that observed by Anthis et *al*. [12] (see Text S1).

Overall, our simulations suggest that optimal association of talin with the membrane is enhanced by conformational flexibility within the head domain, especially between the F0–F1 and F2–F3 pairs. This flexibility facilitates optimal interactions of the V-shaped conformation of talin with the headgroups of anionic lipids. Reduction in the anionic lipid content significantly decreased the talin/bilayer association and, in the presence of mutations that disrupted the talin/lipid electrostatic interactions, talin bound to the bilayer in a perturbed orientation (Fig. S5).

### The Talin Head Domain Perturbs Packing of the Integrin TM Helices

**Figure 6:**
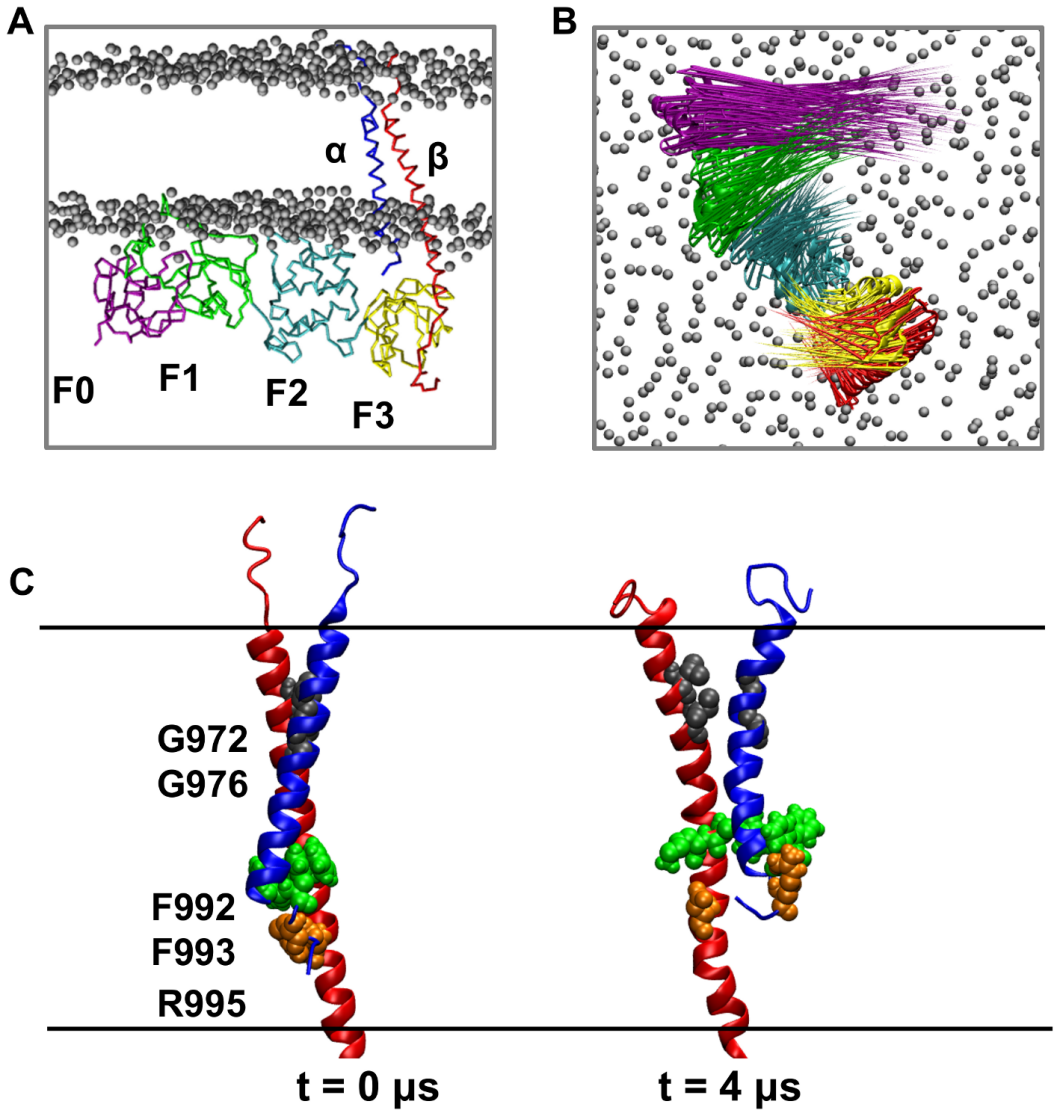
Coarse-grained simulations of a talin/integrin TM complex with a lipid bilayer (simulation *αβ-talh2-CG*; see Table 1). A. The initial state of the *αβ-talh2-CG* simulation. B. Relative movement of the domains of the talin/αβ complex during the *αβ-talh2-CG* simulation. Displacements of all the Cα atoms of the F0 (purple), the F1 (green), the F2 (cyan), the F3 (yellow) and β subunit (red) during the simulation are shown as arrows. The arrows are mapped onto the initial structure and the length of each arrow represents the displacement/direction of the corresponding Cα atom. C. Movement of the integrin TM region observed in the *αβ-talh2-CG* simulation. The β subunit is shown in red and the α subunit in blue. The key residues in the IMC and OMC regions are also shown.

Having established the nature of the interactions of the intact talin head domain with a bilayer, we set out to explore the impact of these interactions on the structure of the integrin TM region. To this end, the talin head domain with optimal bilayer interactions (i.e. from simulation *tal-h2F0-CG*) was modeled in a complex with the α/β integrin TM complex (see Methods for details of modeling this complex). This complex was inserted in a POPC/POPG bilayer (Fig. 6A), and five extended CG-MD simulations (*αβ-talh2-CG*; Table 1) were performed. In these simulations all restraints *between* the integrin α subunit TM domain and the F2–F3/β TM complex were removed to allow the α TM domain to move relative to the rest of the complex. Closer examination of the movement of the individual domains within the complex during the simulations revealed a reorientation of the talin head domain relative to the bilayer surface, comparable to the reorientation observed in our earlier studies of the F2–F3/αβ complex (Fig. 6B). This rotation, in turn, induced a ∼25° rotation of the β TM helix perpendicular to the bilayer normal, similar to that seen in our previous studies (Fig. 6B) [34]. This resulted in the disruption of the interactions in the OMC and IMC regions of the α/β TM segment which were shown to maintain the integrin inactive state [22], [27], [29], [45]–[52]. In particular, the β TM helix rotation perturbed the close packing in the OMC region (because of the 972GxxxG976 motif in the α TM helix) and disrupted the hydrophobic interactions in the IMC region formed by F992 and F993 residues and the αIIb995β3723 salt bridge. Calculation of the angle between the F2–F3 and the F0–F1 domain pairs during the simulations revealed a stable angle of ∼60° (the definition of the angle is the same as described above), indicating that the V-shaped conformation of the talin head was retained in all the simulations (Fig. S7A). We note that simulations of the α/β dimer and the isolated β integrin tails in the same bilayer were performed in our previous study [34].

**Figure 7:**
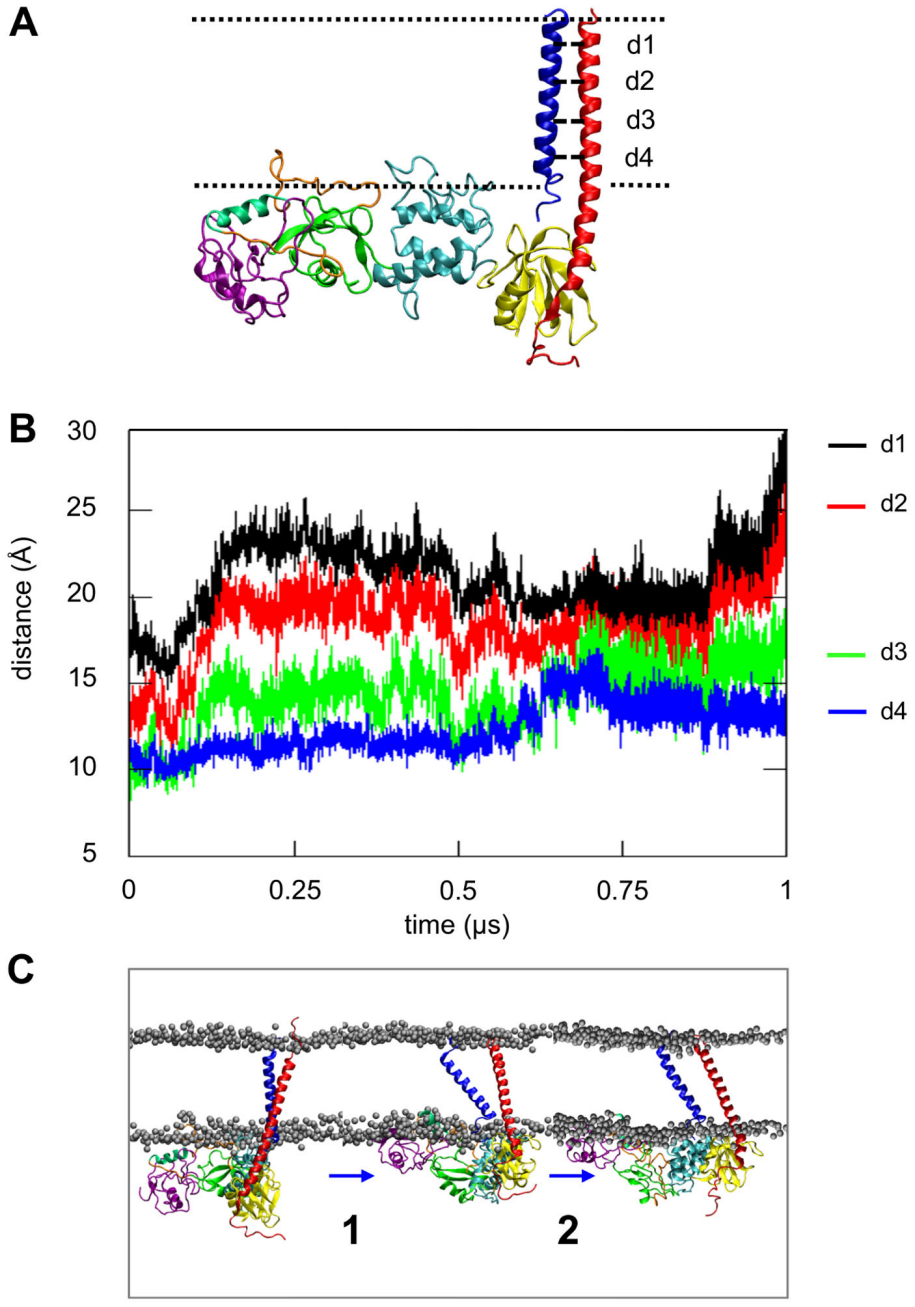
Atomistic simulation of a talin/integrin TM complex with a lipid bilayer (simulation *αβ-talh2o-AT*; see Table 1). A. The talin/integrin TM complex, indicating the four distances (d1 to d4) used to monitor the packing of the TM helices during the simulation. B. Interhelical distances (d1 to d4) as a function of time for the *αβ-talh2o-AT* simulation. Distance d1 shows the separation between the centers of mass of the backbone particles of residues 965–968 of integrin α and residues 694–697 of integrin β as a function of time. Similarly d2, d3 and d4 show the same distances for the α970–973/β700–703, α976-797/β705–708 and α984–987/β714–717 residue groups respectively. Note that all groups are located in the helical region of the integrin TM region. C. Proposed mechanism for the integrin inside-out activation by the bound talin head domain. Electrostatic interactions of talin with negatively charged lipid headgroups promotes reorientation of the talin head domain in a plane perpendicular to the bilayer normal. In turn this rotates the β integrin tail (∼30°) perpendicular to the membrane, disrupting the interactions in both the αIIb/β3 IMC and OMC TM regions. The weakened αIIb/β3 interactions, together with a ∼15° increase in the β TM helix tilt angle relative to the bilayer normal, results in a scissoring movement of the TM regions of the two helices with a modified IMC at the center of the scissors. The scissoring movement is followed by complete dissociation of the two integrin TM helices.

To explore the talin/αβ complex in more detail, a structure which represented an “open” state of the integrin TM domain was selected from the *αβ-talh2-CG* simulation (using similar criteria to those described in our previous simulations of the α/β dimer and the isolated β integrin tails in a bilayer [34]) and converted to an AT representation. In this “open” integrin model, the interactions in both the IMC and OMC regions are disrupted. We have previously suggested that the scissoring motion of integrin TM helices may be “a trigger for inside-out activation” [34]. To explore the consequences of activating this trigger, we performed an extended (microsecond) AT-MD simulation of the complex (*αβ-talh2o-AT*; Table 1). At the end of this atomistic simulation the V-shaped conformation of the talin head domain was retained. Calculation of the inter-helical distances in the OMC and IMC regions revealed an increase in the helix-helix distance in both the IMC and OMC regions (Fig. 7A,B), corresponding to *dissociation* of the α and β TM helices. Dissociation was preceded by a ‘scissoring’ movement similar to that seen in our previous studies of the F2–F3/αβ complex but with more extensive movement of the TM helices and a large increase in the tilt angle of the β helix relative to the bilayer normal, to a final value of 40°, (Fig. S7B). On a similar simulation timescale, the F2–F3 fragment alone induced a much smaller separation of the α/β subunits (Fig. S8B) with a comparable increase in the β TM helix tilt angle to that observed in our previous studies [34]. This suggests that the F2–F3 domains are sufficient to modulate the β tail tilt angle but the entire head is more effective in producing TM helix separation.

**Figure 8:**
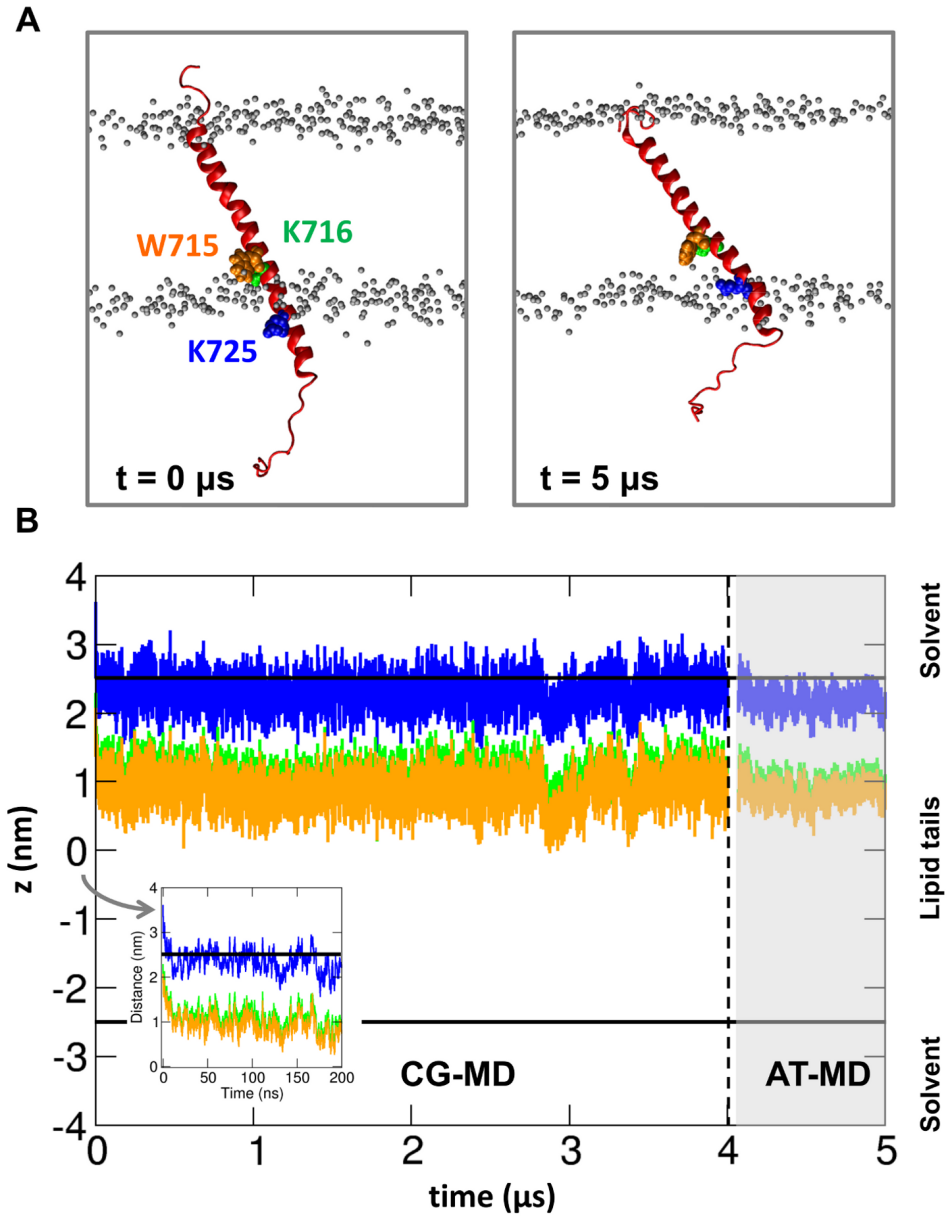
The β-TM helix during simulation of a talin/integrin TM complex with a lipid bilayer (*αβ-talh2-CG* and *αβ-tal2ho-AT* simulations; seeTable 1). A. Position of the β-TM helix at the beginning (t = 0 µs) and at the end (t = 5 µs) of the simulations. Residues W715, K716 and K725 are shown in orange, green and blue respectively. The talin head domain and the α integrin subunit are omitted for clarity, and the lipid bilayer is indicated via the phosphates. B. Positions along the bilayer normal (*z*) during the two simulations of residues W715, K716 and K725 (using the same color code as in A). The horizontal black lines indicate the positions of the phosphates. The inset shows the first 200 ns of the CG simulation. Note that the starting structure for the AT-MD simulations (indicated by a break in the data traces and the vertical broken line) was obtained by merging the trajectories from all five simulations of the *αβ-talh2-CG* simulation ensemble and clustering the TM region of the talin/αβ complex as described in our previous study (see [34]).

The increase in tilt angle induced by the talin head increases the extent of membrane-embedding of the β tail TM region. In the final orientation seen in the simulation with the talin/αβ complex, residues from K716 up to K725 are embedded in the membrane (Fig. 8). A similar increase in the extent of the membrane-embedded region was observed in a recent experimental study [53]. Despite a large increase in β-TM tilt angle, the T715 sidechain remains oriented toward the lipid phosphate atoms, but the K716 sidechain is no longer in contact with the lipid headgroups. This is in good agreement with experimental data that identified interactions between the β3 K716 ε-amine group and the lipid phosphate in the integrin inactive state, suggesting that this interaction controls the tilt angle of the β3 integrin tail [54]. Mutation of K716 in these experimental studies shifted the integrin conformational equilibrium towards an active state, possibly by perturbing the β subunit tilt angle and the α/β TM region crossing angle. Thus, in the active state one would expect weaker K716/lipid headgroup interactions, as observed in our simulations of the integrin active state. The tendency of the β TM helix to adopt a tilted orientation in the membrane is also suggested by other experimental [55] and computational [21] data.

### Conclusions

Our studies show that conformational changes in the talin head on binding to anionic phospholipid membranes mediate transmembrane signaling by the integrin TM helix dimer. Simulations have revealed how optimization of the interactions of the *complete* talin head domain (F0–F3) with an anionic phospholipid bilayer promotes a rearrangement of the subdomains, which in turn initiates a conformational rearrangement of the integrin TM region. In particular, on binding to the membrane the F0–F3 talin head domain undergoes a conformational change to a V-shape, rather than the linear arrangement of the F0-F1-F2-F3 subdomains observed in the crystal structure. Formation of a complex between the rearranged talin head and the integrin TM domain subsequently triggers separation of the TM helices i.e. disassembly of the TM helix dimer. The first stage of this disassembly is an initial scissoring motion of the helices, as seen in our previous studies [34].

The current study reveals how the flexible linker between the F0–F1 and F2–F3 pairs allows talin to adopt a conformation which optimizes its contacts with anionic headgroups of lipids (Fig. 4A). This correlates well with the studies by Bouaouina et al. [8] which have suggested that the activation responses of β3 and β1 integrins have different dependencies on talin head fragments, indicating that formation of an optimal configuration of the talin/membrane complex could also have mechanistic importance.

Our simulations reveal how electrostatic interactions between the protein and anionic lipid headgroups orient the talin head domain and optimize its interactions with the membrane. The cationic surface of the talin head, which binds to the anionic lipids, is formed by the F2 and F3 domains plus the F1 loop. *In silico* mutations (i.e. K324D and K256E, R277E, K272E, R274E) of this surface perturb binding of talin to the membrane, in agreement with experimental studies [12], [17], [20], [21]. This binding surface is consistent with NMR [10], crystallographic [12] and TIRF microscopy studies [21].

Experimental studies indicate that inserted loop in the F1 domain is dynamic in nature [17], [20]. Our simulations suggest that it provides a binding surface close to the cationic surface of F2–F3. In all simulations that yielded a ‘productive’ orientation of the talin head domain on the membrane, the F2–F3 pair always associated prior to F0–F1. This agrees with recent data that the association constant of the complete talin head domain with lipids is similar to that of the F2–F3 fragment [56]. We note that in kindlin, a homolog of talin that co-activates integrins, an even longer lysine-rich loop is inserted in the F1 domain. This lysine rich loop in kindlin is highly conserved and is believed to support binding to anionic phospholipid head groups [57].

Simulations that included the entire talin head/membrane/TM complex revealed that talin interactions with the integrin β tail and the membrane surface disrupt the interactions of both the OMC and IMC regions, resulting in eventual dissociation of the TM helices. Calculation of the hydrogen bonds between the lipids and the membrane (see Fig. S7C) after the disruption of interactions in the TM region show an increase in the number of lipid/talin hydrogen bonds. This suggests a stronger association between talin and the bilayer after formation of the talin/αβ complex. This could explain why the intact talin head domain has more dramatic effect than F2–F3 alone. The crystal structure of the integrin ectodomain and cysteine disulfide mapping of an intact integrin [58], [59] suggest that the α and β domains are in close proximity to one another in the inactive state. Thus a scissoring movement followed by dissociation of the two TM helices provides a plausible model for how the TM domain may trigger a conformational change in the ectodomain leading subsequent adoption of an extended active state.

From a more general perspective, this study reveals the interplay of membrane interactions and conformational changes involved in transmembrane signaling by receptors and associated proteins and/or domains. In particular, it may be compared with recent simulation studies, e.g. of the EGF receptor [42], which suggested that substantive repacking of the TM helix dimer and interactions between the intracellular kinase domain and anionic lipids play a key role in signaling across a membrane. The mechanisms in these two classes of membrane receptors (i.e. integrins and receptor tyrosine kinases respectively) may be compared with movements of TM helices thought to mediate signaling in GPCRs (as revealed by crystallographic, NMR and simulation studies [60]). One possible consequence of the extensive movements of TM helices is that signaling mechanisms are likely to be modulated by changes in (local) lipid bilayer properties [61]. This clearly merits further investigation by both computational and biophysical approaches.

## Methods

### CG-MD Simulations

The CG-MD simulations were performed using a local variant [31], [62] of the MARTINI forcefield [32]. A mapping of approximately 4∶1 heavy atoms to CG particles was used. Harmonic restraints (i.e. an elastic network model; ENM) between backbone particles within a cut-off distance of 7 Å was applied with a harmonic restraint force constant of 10 kJ/mol/Å^2^. In the *tal-l25-CG* and *tal-l50-CG* simulations (Table S1) the force constant for the ENM was set to 25 kJ/mol/Å^2^ and 50 kJ/mol/Å^2^ respectively and the cut-off distance was increased to 10 Å. The bilayer was constructed by self-assembly CG-MD simulations. In these simulations the lipids were placed randomly within a simulation box and solvated with CG water molecules and ions to neutralize the system. Subsequently, a production simulation was performed for 200 ns. After the first 10–15 ns of simulation the bilayer formed with an equal distribution of lipids in the two leaflets. For the simulation systems discussed here, two different bilayers were constructed. The first bilayer contained 832 zwitterionic POPC lipids and the second had 512 POPC and 320 POPG lipids (ratio of 3∶2). In the CG simulations the center of mass of the protein was placed 120 Å from the center of mass of the preformed bilayer (Fig. S3A). This starting distance between protein and bilayer was chosen to be much larger than the cut-off distance used for the electrostatic and the van der Waals terms in the CG forcefield. All systems were subsequently solvated with CG water molecules and neutralized with CG sodium particles, energy minimized for 250 steps and equilibrated for 5 ns with the protein Cα particles restrained (force constant 10 kJ/mol/Å^2^). Finally, CG-MD simulations were performed. The final snapshot of the CG-MD simulation was converted to an atomistic (AT) representation, using a fragment-based approach [33], for further refinement.

For the simulations with the talin/αβ complex, the same POPC/POPG bilayer as above was used. The TM region of the talin/αβ complex was inserted in the bilayer using GROMACS. The lipids that overlapped with the integrin TM region were removed. The same energy minimization and equilibration steps were performed as described above. A modified CG model was used where all the ENM restraints between the integrin α TM region and the rest of the complex were removed.

All CG-MD simulations were performed using GROMACS 4.5 (www.gromacs.org) [63], [64]. A Berendsen thermostat [65] was used for temperature coupling with a coupling constant of 1.0 ps and a reference temperature of 310 K. The Lennard-Jones and Coulombic interactions were shifted to zero between 9 Å and 12 Å, and 0 to 12 Å respectively. The time step was 20 fs. A Berendsen barostat was used for pressure coupling. The coupling constant was 1.0 ps, the compressibility was 5.0×10^−6^ bar^−1^ and the reference pressure was 1 bar.

### Atomistic Molecular Dynamics (AT-MD) Simulations

The AT-MD simulations were performed using the GROMOS96 43a1 forcefield [66]. The Parrinello-Rahman barostat [67] and the Berendsen thermostat [65] were used for pressure and temperature coupling, respectively. The bond length was constrained using the LINCS algorithm [68] and the particle mesh Ewald (PME) algorithm [69] was used to model long-range electrostatic interactions. A cut-off distance of 10 Å was used for the van der Waals interactions. All the AT simulation systems were energy minimized using a steepest descent algorithm and equilibrated for 2.5 ns with the protein Cα atoms restrained (force constant 10 kJ/mol/Å^2^). Subsequently, unrestrained AT-MD simulations were performed. All the analyses were performed using GROMACS (www.gromacs.org) [63], [64], VMD [70] and locally written codes.

## Supporting Information

Figure S1 The compete talin head domain. A. Positions of the F1 loop (in different conformations) relative to the rest of the talin head domain at the beginning of the CG-MD simulations. See Tables 1 and S1. B. Sequence of the talin head domain. The F3 domain is shown in yellow, the F2 domain in cyan, the F1 domain in green, the F1 insertion in orange and the F0 domain in purple. The proposed helical regions in the F1 loop are also shown (h1 and h2).
(PDF)

Figure S2 Electrostatic profile of the talin head domain. Surface electrostatic potential representation for the crystal structure of the talin head domain (above) and the final snapshot of the *tal-sol-AT* simulation (below). In both cases the surface shown to interact with the bilayer is oriented towards the reader. The electrostatic calculation was performed using APBS [71] in PyMol [72]. The electrostatic potential ranges from −0.5 kT/e (red) to +0.5 kT/e (blue).
(PDF)

Figure S3 Association of the talin head domain with a lipid bilayer. A. Progress of the simulations with the complete talin head domain. Snapshots from the *tal-h2F0-CG* simulation at 0 ns, 900 ns and 1.5 µs. The color scheme for the talin domains is the same as in Fig. 1. The lipids are shown in grey and waters are omitted for clarity. B. Distance between the centers of mass of talin and a lipid bilayer as a function of time for the simulation *tal-h2F0pc-CG* with a zwitterionic (POPC) lipid bilayer. The different colored lines correspond to the five repeat simulations. The horizontal broken line indicates the distance when talin is associated with the bilayer surface.
(PDF)

Figure S4 Interactions between the talin F0 subdomain and the h2 helix. A,B. Normalized average number of contacts (across all the *tal-h2F0-AT* simulations) between the talin F0 domain (A) and the h2 helix (B). The contacts are mapped on the F0 and h2 helix structures. Blue indicates a low number, white indicates a medium number and red a large number of contacts. Contacts are defined by using a distance cut-off of 3.5 Å between the F0 residues and the h2 helix residues.
(PDF)

Figure S5 Simulations with experimentally tested mutations perturb the orientation of talin head domain relative to the bilayer. A i and ii. Separation between the centers of mass of talin and the bilayer as a function of time for the *tal-l4E-CG* and *tal-lK324D-CG* simulations (see Table S1). B. Snapshot demonstrating the final orientation of the talin head domain in the *tal-lK324D-CG* simulation with the K324D mutation in the F3 positively charged loop. The color scheme is the same as in other Figures with the backbone particle of the mutated residue shown as a green sphere. The binding surface of the F2 and F3 domains, identified earlier, which positions the talin F3 domain in an orientation that would facilitate formation of a complex similar to the known structure of the F3/β-integrin tail complex is shown as a light red surface.
(PDF)

Figure S6 Comparison of the interaction between the talin head domain and the POPC and POPG lipids. A,B. Number of hydrogen bonds between the protein and POPC (black) and POPG lipids (red) for the *tal-AT* (A) and the *tal-h2F0-AT* (B) simulations.
(PDF)

Figure S7 Atomistic simulations of the talin/integrin complex in a lipid bilayer. A. Movement of the F0–F1 pair relative to the F2–F3 pair as a function of time for the three AT systems with the talin head domain. Inset pictures show the talin conformation for each case. B. The β helix tilt angle relative to the bilayer normal as a function of time for the *αβ-talh2o-AT* (red), the *αβ-talh2p-AT* (green) and the *αβ-tal-AT* (black) simulations. C. Number of H-bonds between the talin and the lipids (shown separated for POPC (black), POPG (red) and all (orange) lipids) as a function of time for the *αβ-talh2o-AT* simulation.
(PDF)

Figure S8 Talin facilitates rearrangement of the integrin TM region. A. Scissoring movement of the integrin TM region helices. The scissoring movement is shown at 100 ns, 200 ns and 1 µs for the *αβ-talh2o-AT* simulation (see Table S2 for more information). The F2–F3/αβ complex (left) is also shown for comparison. The α integrin complex is shown in blue, the F0 (purple), the F1 (green), the F2 (cyan), the F3 (yellow) and the β subunit (red). Black lines indicate the lipid phosphate atoms. Note that in this orientation the large tilt of the β-TM helix is not observed. B. Inter-helical distances (d1 to d4) as a function of time for the simulation with the F2–F3/αβ complex. The same regions as in Fig. 7A are used.
(PDF)

Table S1 Details of all talin simulations. This table provides details of all the talin simulations performed in this study.
(PDF)

Table S2 Details of all talin/integrin TM simulations. This table provides details of all the simulations of the talin/integrin TM complex performed in this study.
(PDF)

Table S3 Important residues for the talin/lipid interactions. This table provides details for all the residues that made more than 90% of the interaction with the lipids in our simulations.
(PDF)

Text S1 Supplementary results from control and sensitivity analysis simulations. This text provides results from a number of control simulations and simulations evaluating the sensitivity of the main results to changes in simulation setup, in particular for: (i) the relative orientation of F0–F1 and F2–F3 pairs and role of F1 loop; (ii) the role of the negatively charge lipids in talin/membrane interactions; and (iii) the talin/αβ complex.
(PDF)
