# Supplementary figures and images for: Conformational Changes in Talin on Binding to Anionic Phospholipid Membranes Facilitate Signaling by Integrin Transmembrane Helices
Source: PLoS Comput Biol. 2013 Oct 31;9(10):e1003316. doi: 10.1371/journal.pcbi.1003316 (PMC3814715; doi:10.1371/journal.pcbi.1003316)

Figure S1:

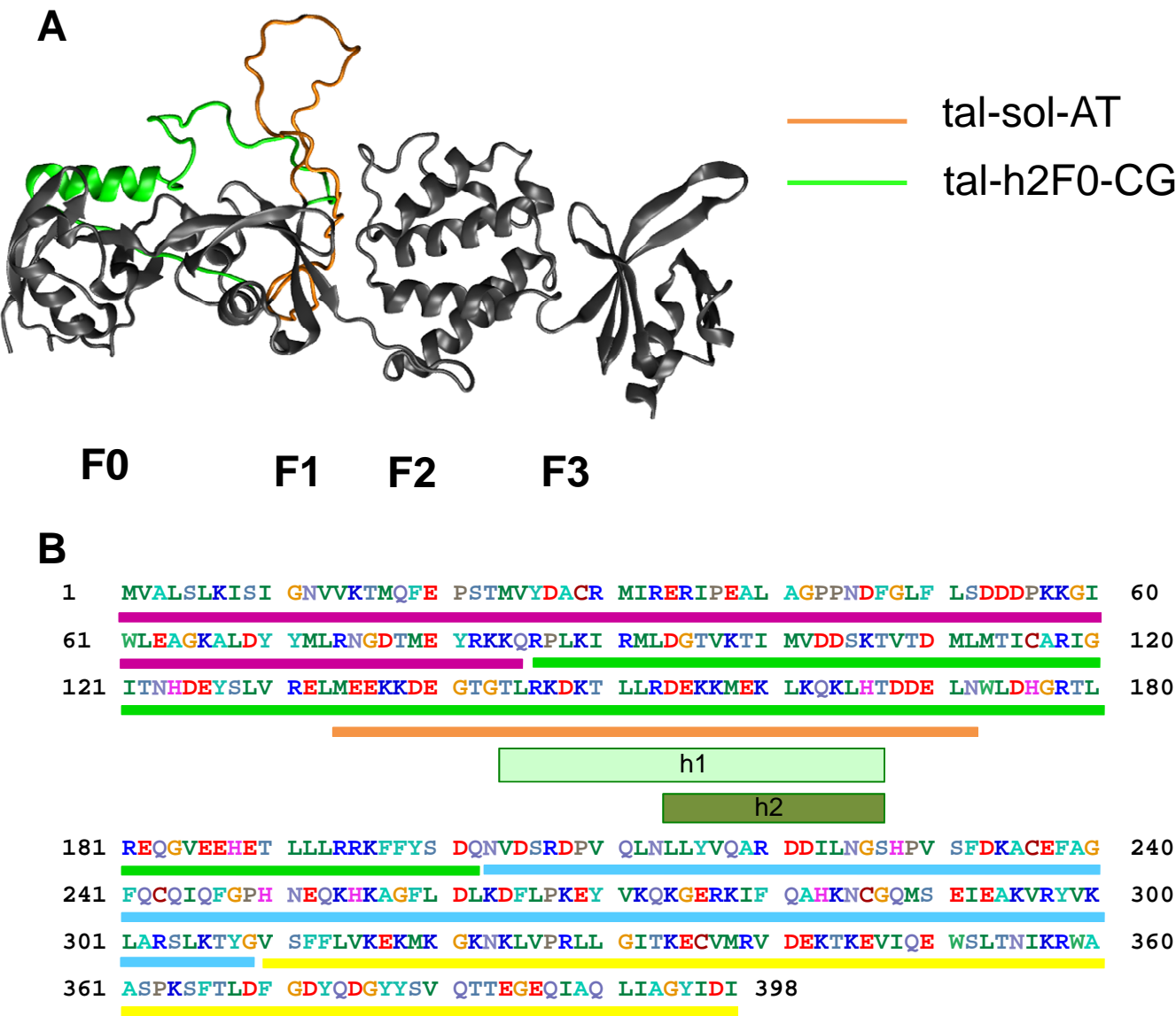

Supplement: Figure S1 — The compete talin head domain. A. Positions of the F1 loop (in different conformations) relative to the rest of the talin head domain at the beginning of the CG-MD simulations. See Tables 1 and S1. B. Sequence of the talin head domain. The F3 domain is shown in yellow, the F2 domain in cyan, the F1 domain in green, the F1 insertion in orange and the F0 domain in purple. The proposed helical regions in the F1 loop are also shown (h1 and h2). (PDF) [file pcbi.1003316.s001.pdf]

Figure S2:

**Crystal structure:**

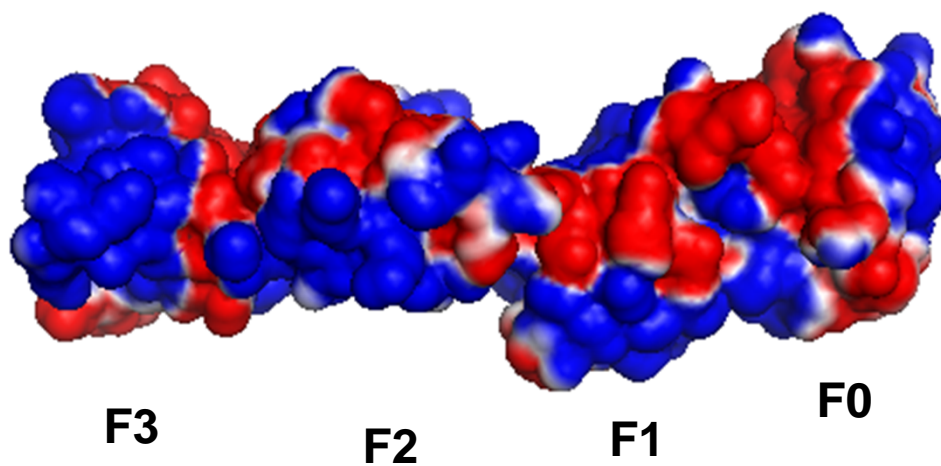

**Final snapshot from the tal\_sol-AT simulation:**

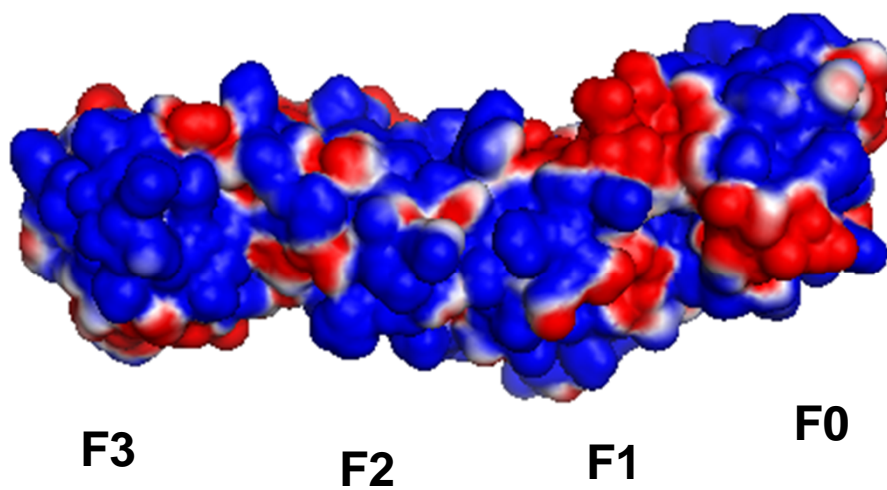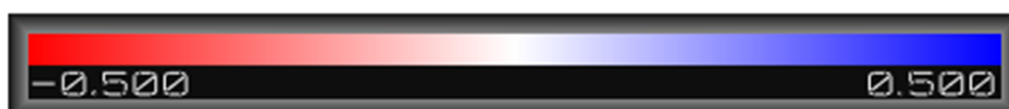

Supplement: Figure S2 — Electrostatic profile of the talin head domain. Surface electrostatic potential representation for the crystal structure of the talin head domain (above) and the final snapshot of the tal-sol-AT simulation (below). In both cases the surface shown to interact with the bilayer is oriented towards the reader. The electrostatic calculation was performed using APBS [71] in PyMol [72]. The electrostatic potential ranges from −0.5 kT/e (red) to +0.5 kT/e (blue). (PDF) [file pcbi.1003316.s002.pdf]

Figure S3:

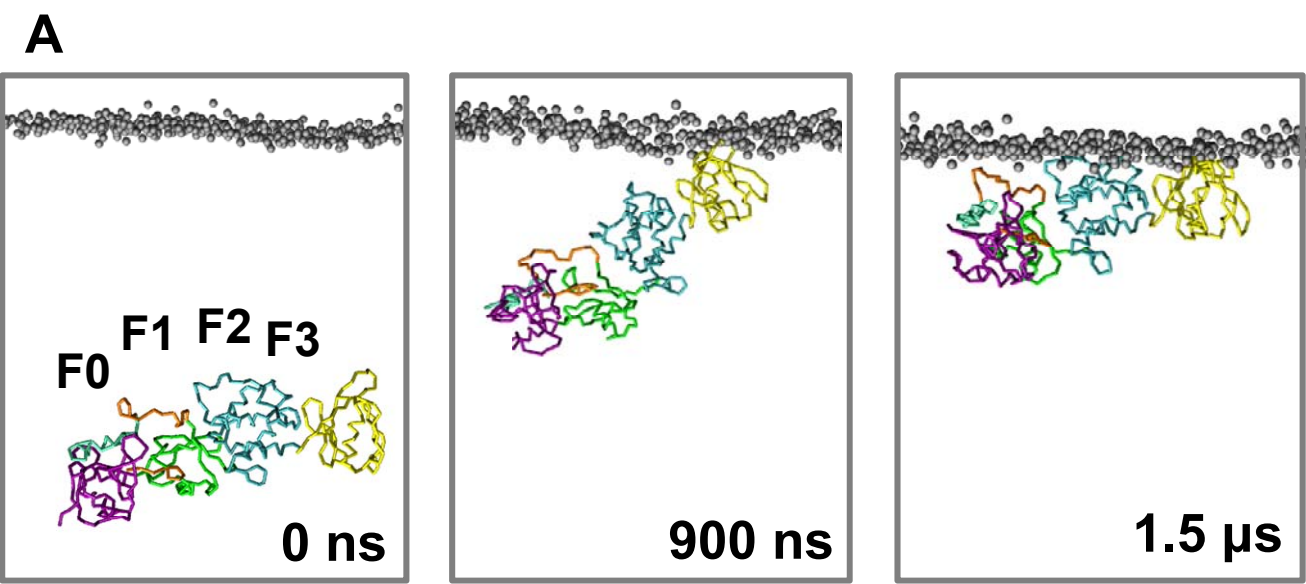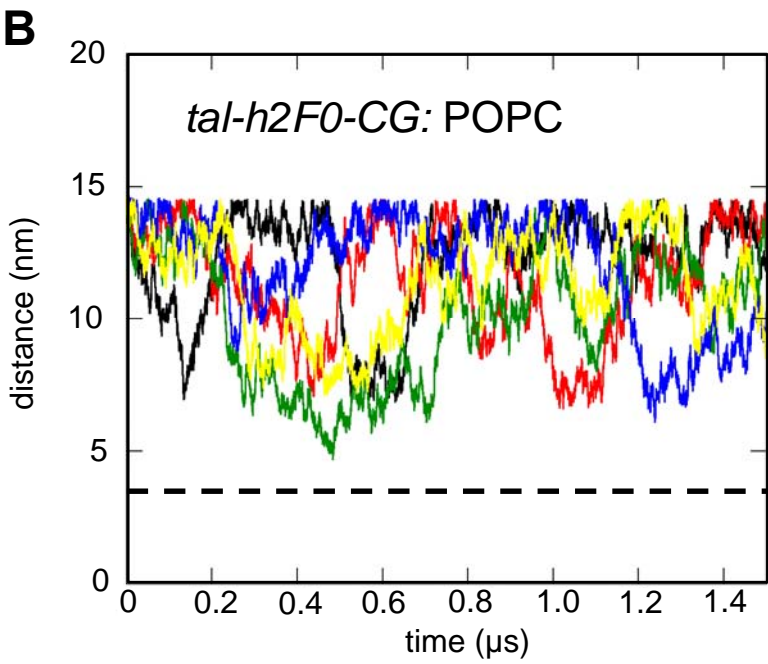

Supplement: Figure S3 — Association of the talin head domain with a lipid bilayer. A. Progress of the simulations with the complete talin head domain. Snapshots from the tal-h2F0-CG simulation at 0 ns, 900 ns and 1.5 µs. The color scheme for the talin domains is the same as in Fig. 1. The lipids are shown in grey and waters are omitted for clarity. B. Distance between the centers of mass of talin and a lipid bilayer as a function of time for the simulation tal-h2F0pc-CG with a zwitterionic (POPC) lipid bilayer. The different colored lines correspond to the five repeat simulations. The horizontal broken line indicates the distance when talin is associated with the bilayer surface. (PDF) [file pcbi.1003316.s003.pdf]

Figure S4:

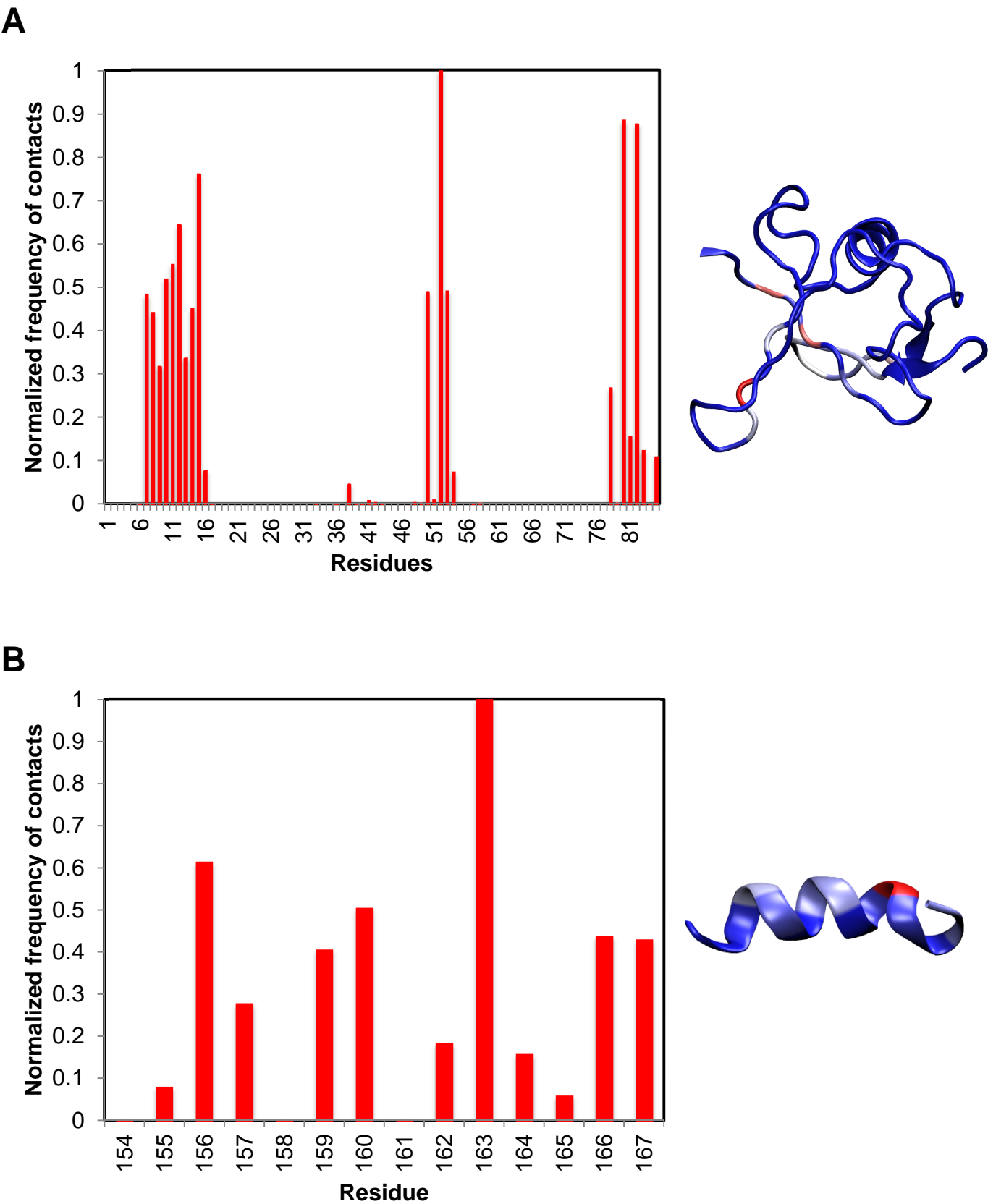

Supplement: Figure S4 — Interactions between the talin F0 subdomain and the h2 helix. A,B. Normalized average number of contacts (across all the tal-h2F0-AT simulations) between the talin F0 domain (A) and the h2 helix (B). The contacts are mapped on the F0 and h2 helix structures. Blue indicates a low number, white indicates a medium number and red a large number of contacts. Contacts are defined by using a distance cut-off of 3.5 Å between the F0 residues and the h2 helix residues. (PDF) [file pcbi.1003316.s004.pdf]

Figure S5:

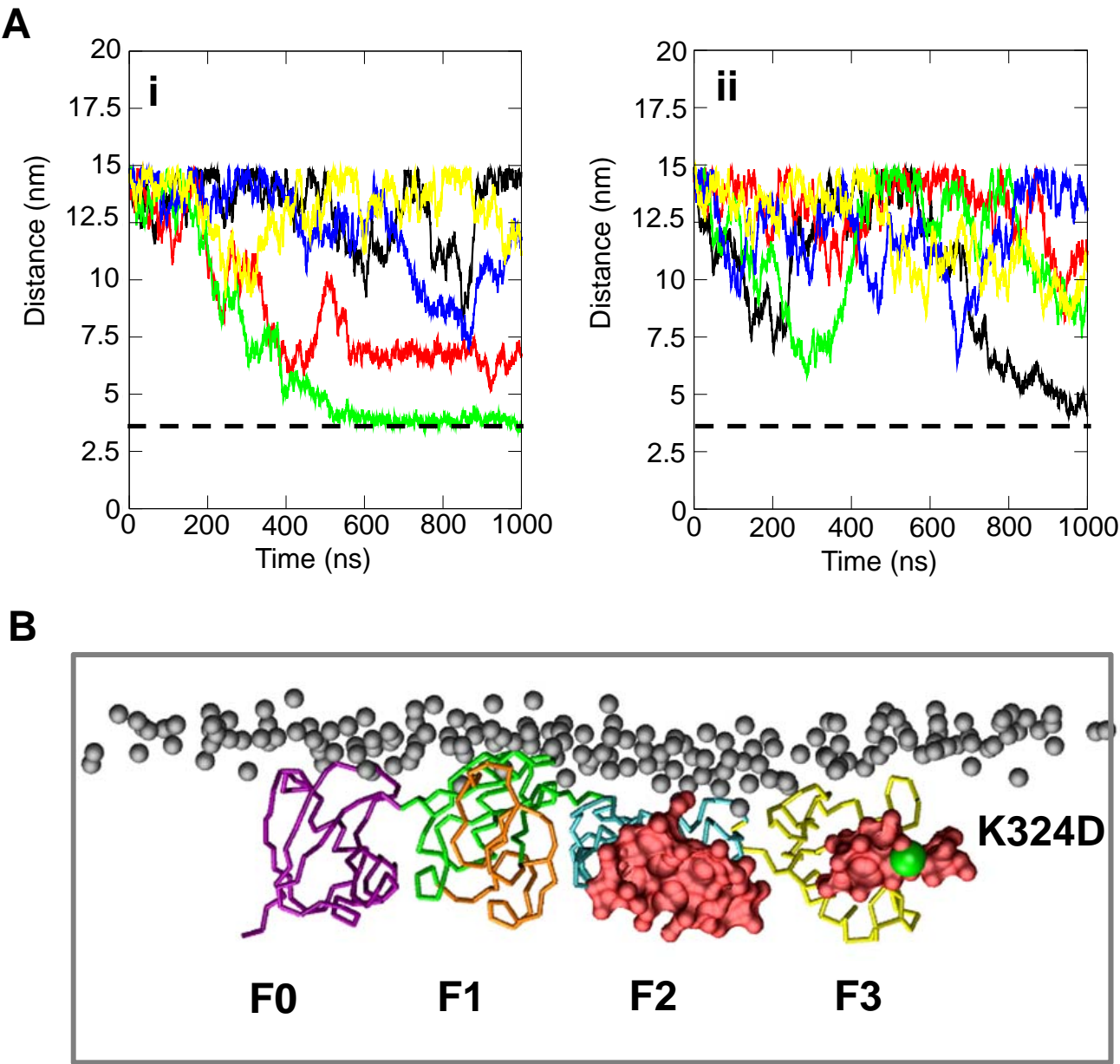

Supplement: Figure S5 — Simulations with experimentally tested mutations perturb the orientation of talin head domain relative to the bilayer. A i and ii. Separation between the centers of mass of talin and the bilayer as a function of time for the tal-l4E-CG and tal-lK324D-CG simulations (see Table S1). B. Snapshot demonstrating the final orientation of the talin head domain in the tal-lK324D-CG simulation with the K324D mutation in the F3 positively charged loop. The color scheme is the same as in other Figures with the backbone particle of the mutated residue shown as a green sphere. The binding surface of the F2 and F3 domains, identified earlier, which positions the talin F3 domain in an orientation that would facilitate formation of a complex similar to the known structure of the F3/β-integrin tail complex is shown as a light red surface. (PDF) [file pcbi.1003316.s005.pdf]

Figure S6:

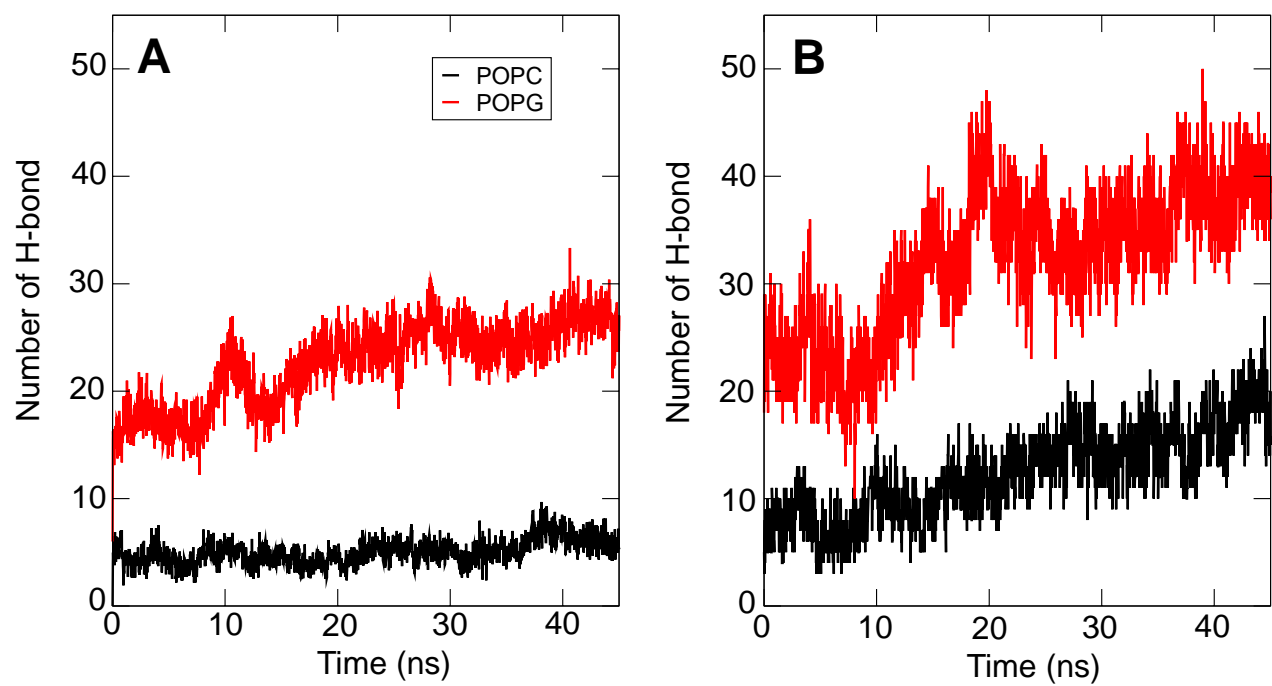

Supplement: Figure S6 — Comparison of the interaction between the talin head domain and the POPC and POPG lipids. A,B. Number of hydrogen bonds between the protein and POPC (black) and POPG lipids (red) for the tal-AT (A) and the tal-h2F0-AT (B) simulations. (PDF) [file pcbi.1003316.s006.pdf]

Figure S7:

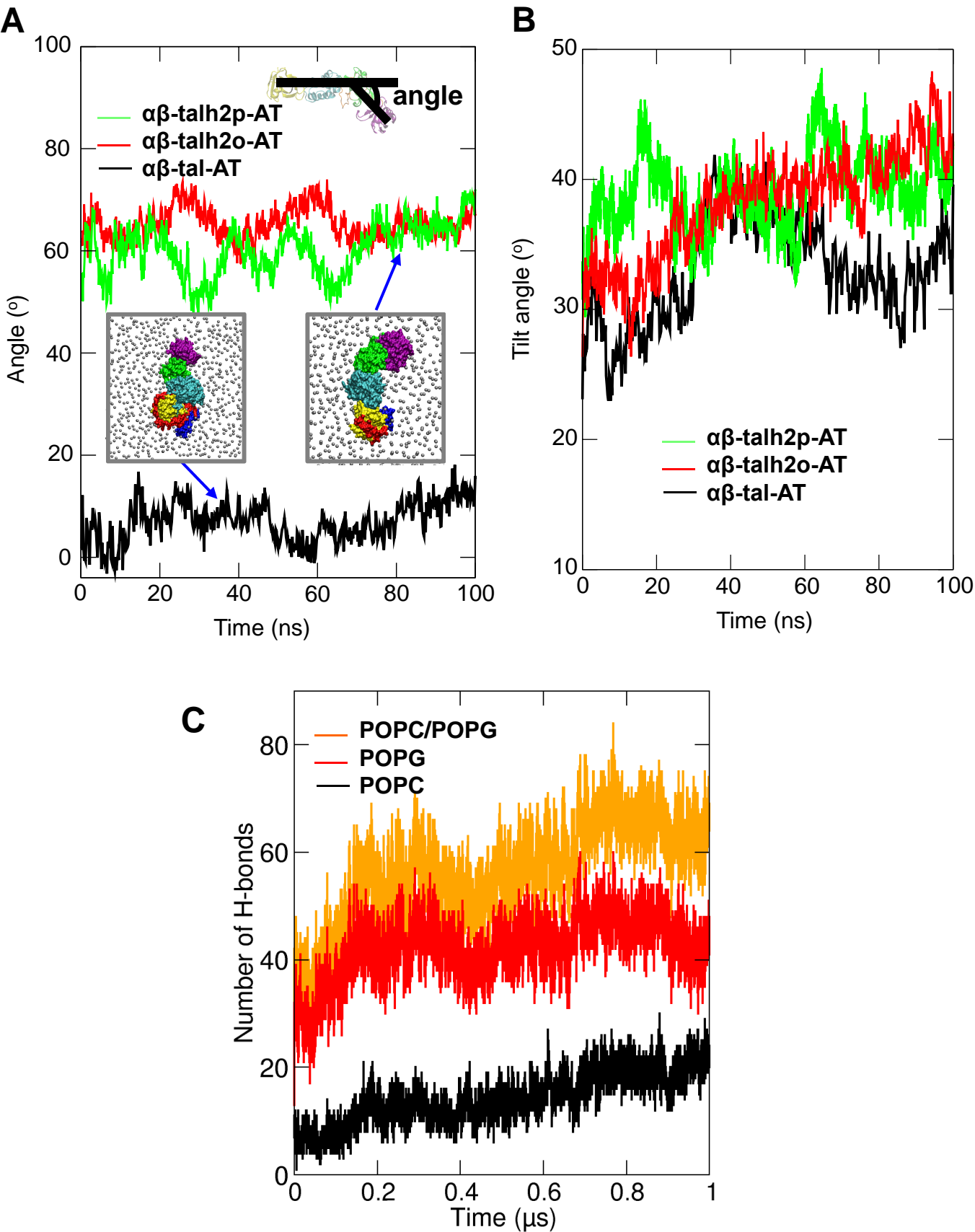

Supplement: Figure S7 — Atomistic simulations of the talin/integrin complex in a lipid bilayer. A. Movement of the F0–F1 pair relative to the F2–F3 pair as a function of time for the three AT systems with the talin head domain. Inset pictures show the talin conformation for each case. B. The β helix tilt angle relative to the bilayer normal as a function of time for the αβ-talh2o-AT (red), the αβ-talh2p-AT (green) and the αβ-tal-AT (black) simulations. C. Number of H-bonds between the talin and the lipids (shown separated for POPC (black), POPG (red) and all (orange) lipids) as a function of time for the αβ-talh2o-AT simulation. (PDF) [file pcbi.1003316.s007.pdf]

Figure S8:

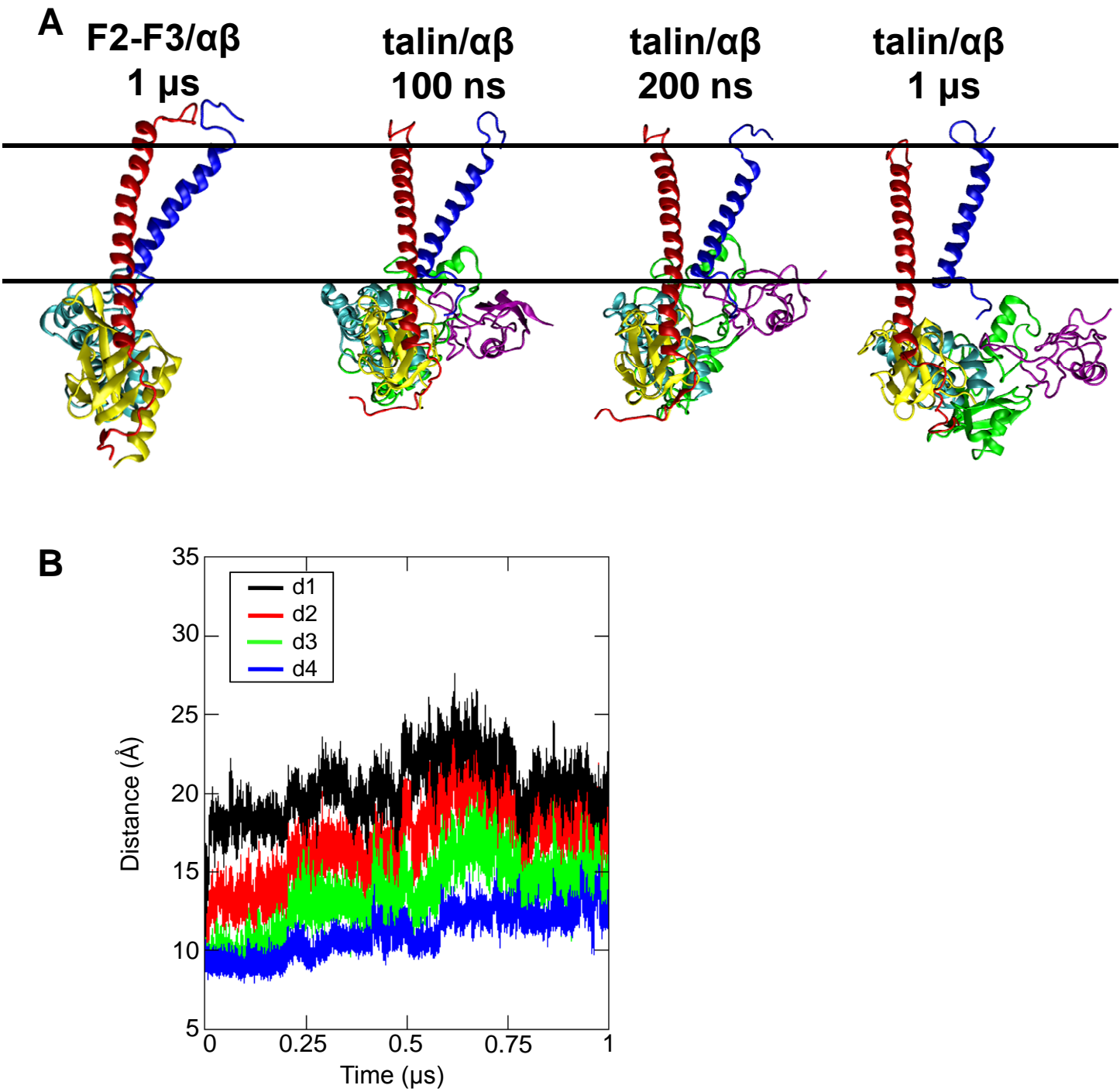

Supplement: Figure S8 — Talin facilitates rearrangement of the integrin TM region. A. Scissoring movement of the integrin TM region helices. The scissoring movement is shown at 100 ns, 200 ns and 1 µs for the αβ-talh2o-AT simulation (see Table S2 for more information). The F2–F3/αβ complex (left) is also shown for comparison. The α integrin complex is shown in blue, the F0 (purple), the F1 (green), the F2 (cyan), the F3 (yellow) and the β subunit (red). Black lines indicate the lipid phosphate atoms. Note that in this orientation the large tilt of the β-TM helix is not observed. B. Inter-helical distances (d1 to d4) as a function of time for the simulation with the F2–F3/αβ complex. The same regions as in Fig. 7A are used. (PDF) [file pcbi.1003316.s008.pdf]
