# Supplementary material for: Conformational Changes in Talin on Binding to Anionic Phospholipid Membranes Facilitate Signaling by Integrin Transmembrane Helices
Source: PLoS Comput Biol. 2013 Oct 31;9(10):e1003316. doi: 10.1371/journal.pcbi.1003316 (PMC3814715; doi:10.1371/journal.pcbi.1003316)
Supplement: Table S1 — Details of all talin simulations. This table provides details of all the talin simulations performed in this study. (PDF) [file pcbi.1003316.s009.pdf]

Table S1: Details of All Talin Simulations

Simulation	Proteins	Duration (ns)
tal-sol-AT	F0-F1-F2-F3 (loop-res: 134-172; simulation in aqueous solution)	2x100
tal-h2F0-CG	F0-F1-F2-F3 (loop-res: 134-172/helix-res:154-167; conformation derived from the tal-sol-AT simulations)	5x1500
tal-h2F0pc-CG	F0-F1-F2-F3 (loop-res: 134-172/helix-res:154-167; conformation derived from the tal-sol-AT simulations)	5x1500
tal-lF0F1-CG	F0-F1-F2-F3 (loop-res: 134-172; conformation derived from the tal-sol-AT simulations)	5x1500
tal-l25-CG	F0-F1-F2-F3 (loop-res: 134-172; harmonically restrained 25 kJ/mol/ Å^2])	5x1000
tal-l50-CG	F0-F1-F2-F3 (loop-res: 134-172; harmonic restrained 50 kJ/mol/ Å^2])	5x1000
tal-l4E-CG	F0-F1-F2-F3 (loop-res: 134-172 [4E])	5x1000
tal-lK324D-CG	F0-F1-F2-F3 (loop-res: 134-172 [K324D])	5x1000
tal-h2F0-AT	F0-F1-F2-F3 (loop-res: 134-172/helix-res:154-167)	3x45

In all simulations a POPC/POPG bilayer (ratio 3:2) was used, with the exception of the *tal-h2F0pc-CG* where a POPC bilayer was used.
