# Supplementary material for: Conformational Changes in Talin on Binding to Anionic Phospholipid Membranes Facilitate Signaling by Integrin Transmembrane Helices
Source: PLoS Comput Biol. 2013 Oct 31;9(10):e1003316. doi: 10.1371/journal.pcbi.1003316 (PMC3814715; doi:10.1371/journal.pcbi.1003316)
Supplement: Table S2 — Details of all talin/integrin TM simulations. This table provides details of all the simulations of the talin/integrin TM complex performed in this study. (PDF) [file pcbi.1003316.s010.pdf]

Table S2: Details of all talin/integrin TM simulations.

Simulation	Proteins	Coordinates	Duration (ns)
αβ-talh2-CG	αIIb/β* TM/talin (loop-res: 134-172/helix-res: 154-167)	2K9J + 3G9W+ 3IVF	5x4000
αβ-talh2p-AT	αIIb/β* TM/talin (loop-res: 134-172/helix-res: 154-167) partial		3x100
αβ-talh2o-AT	αIIb/β* TM/talin (loop-res: 134-172/helix-res: 154-167) open		2x100, 1x1000

TM = transmembrane helix. β* indicates a chimeric β chain (β3/β1D) – see Methods for details). All simulations were of the protein complex in a POPC/POPG lipid bilayer.
