# Supplementary material for: Conformational Changes in Talin on Binding to Anionic Phospholipid Membranes Facilitate Signaling by Integrin Transmembrane Helices
Source: PLoS Comput Biol. 2013 Oct 31;9(10):e1003316. doi: 10.1371/journal.pcbi.1003316 (PMC3814715; doi:10.1371/journal.pcbi.1003316)
Supplement: Table S3 — Important residues for the talin/lipid interactions. This table provides details for all the residues that made more than 90% of the interaction with the lipids in our simulations. (PDF) [file pcbi.1003316.s011.pdf]

Domain	Residue Type	Residue Number
F0	LYS	15
	THR	16
	ASP	53
	ASP	54
F1	LYS	137
	GLU	140
	THR	144
	LEU	145
	ARG	146
	LYS	147
	LEU	165
	HIS	166
	THR	167
F2	GLU	252
	GLN	253
	LYS	254
	LYS	256
	PHE	259
	LYS	263
	ASP	264
	GLU	269
	LYS	272
	GLN	273
	LYS	274
	ARG	277
	PHE	280
F3	LYS	318
	MET	319
	GLY	321
	LYS	322
	LEU	325
	VAL	326
	GLU	342
	LYS	343
	LYS	364
	GLN	381
	THR	383
