# Supplementary material for: Conformational Changes in Talin on Binding to Anionic Phospholipid Membranes Facilitate Signaling by Integrin Transmembrane Helices
Source: PLoS Comput Biol. 2013 Oct 31;9(10):e1003316. doi: 10.1371/journal.pcbi.1003316 (PMC3814715; doi:10.1371/journal.pcbi.1003316)
Supplement: Text S1 — Supplementary results from control and sensitivity analysis simulations. This text provides results from a number of control simulations and simulations evaluating the sensitivity of the main results to changes in simulation setup, in particular for: (i) the relative orientation of F0–F1 and F2–F3 pairs and role of F1 loop; (ii) the role of the negatively charge lipids in talin/membrane interactions; and (iii) the talin/αβ complex. (PDF) [file pcbi.1003316.s012.pdf]

Text S1: Supplementary Results from Control and Sensitivity Analysis Simulations

Relative orientation of F0-F1 and F2-F3 pairs and role of F1 loop:

The simulation with the talin head domain in solution (*tal-sol-AT* in Table S1; see main text) suggested a tendency for the F1 loop to be close to the F0-F1 on the same side as the positively charged surfaces on F2 and F3 (Fig. 3). Restricting the loop conformation to have a similar form (but without the helical component suggested by NMR data as described in the main text), five simulations (*tal-lF0F1-CG*) were also initiated. In this case only three out of five simulations resulted in a talin/bilayer complex compared to four out of five in the *tal-h2F0-CG* simulation (see main text), suggesting that the formation of a small helical region close to the F0 domain prior to binding to the bilayer slightly increased the talin/lipid association. Similar to the *tal-h2F0-CG* simulation (see main text) talin bound to the bilayer in the same orientation and adopted the same V-shaped configuration. Transient variation in angle between the F0-F1 and F2-F3 domain pairs was also observed when talin was in solution but the V-shaped conformer was stabilized by association with the bilayer. The residues described in Table S3 again made the largest number of contacts with the lipids in the bilayer.

The second important structural feature of talin suggested by the above simulations is that the optimum association of talin with the membrane is enhanced by flexibility within the molecule especially between the F2-F3 and F0-F1 domain pairs. To further examine the role of interdomain linkers, a coarse-grained model of talin with the loop modeled was prepared where the force constant and the cut-off distance of the ENM network (see methods) were increased. This essentially means that no flexibility between the domains was allowed during the simulations. Similar to the simulations above, the protein with the modified ENM network was positioned away from a preformed POPC/POPG bilayer (*tal-l25-CG* and *tal-l50-CG* in Table S1). Interestingly, in none of these simulations talin bound to the bilayer in a way that would facilitate binding of F2-F3 to the β-tail. In all simulations the lack of flexibility prevented the association of the F2-F3 domain with the bilayer before the F0-F1/bilayer association. These results again suggest that flexibility between the talin subdomains is needed for optimal talin/membrane and talin/integrin interactions. In all simulations the final orientation of talin relative to the bilayer was similar to that observed for the mutated forms of talin (see below) as shown in Fig. S5A.

The role of the negatively charge lipids in talin/membrane interactions:

All the simulations described in the main text highlighted the crucial role of negatively charged lipids in the binding and interactions of the talin head domain with the membrane. To test this

observation further, similar simulations to the *tal-F0h2-CG* system (i.e. with the loop in an elongated form and the h2 helix modelled on the loop) were set up where the POPC/POPG bilayer used earlier was replaced by a pure POPC bilayer (i.e. with a net zero charge). In good agreement with previous observations, talin remained in the aqueous environment in all five simulations without forming a complex with the bilayer. Similar to the *tal-F0h2-CG* system a transient displacement of the F0-F1 pair relative to the F2-F3 pair was observed. Furthermore, additional simulations using the same POPC/POPG bilayer as above (see main text) and the talin head domain with the loop modeled in a random configuration were set up with mutations in the F3 acidic loop (*tal-K324D-CG*) or the F2 positively charged patch (*tal-l4E-CG*). Experimental studies have suggested that these mutations reduce the affinity of the talin head domain for the membrane and reduce integrin activation. The simulations with the mutated form of talin are in good agreement with these studies since in both simulation systems the talin head domain bound to the bilayer with reduced affinity (one out of five simulations in the *tal-l4E-CG* and two out of five simulations in the *tal-K324D-CG*; Fig. S5A). In all simulations in which the talin/bilayer complex was formed, talin bound to the bilayer in a perturbed orientation, similar to that shown in Fig. S5B.

The talin/αβ complex:

In addition to the “open” state of integrins which is described in the main text a structure which represents a “partially” disrupted state was selected from the *αβ-talh2-CG* simulation using similar criteria to that described previously and converted to AT representation. In the “partially” disrupted case the IMC interactions remained intact whereas the interactions in the OMC region were disrupted. At the end of the simulations there was no significant movement between the two helices despite a comparable increase in the β integrin TM region tilt angle during the simulations (~40°; see Fig. S7B). In this case the interactions of the aromatic cluster (F992, F993) with the β integrin W715 and K716 residues were retained throughout the simulation. The orientation of talin relative to the bilayer and its V-shape configuration were retained during the simulations.
